# Tetrapod tracks in Permo–Triassic eolian beds of southern Brazil (Paraná Basin)

**DOI:** 10.7717/peerj.4764

**Published:** 2018-05-18

**Authors:** Heitor Francischini, Paula Dentzien-Dias, Spencer G. Lucas, Cesar L. Schultz

**Affiliations:** 1 Laboratório de Paleontologia de Vertebrados, Instituto de Geociências, Universidade Federal do Rio Grande do Sul, Porto Alegre, Rio Grande do Sul, Brazil; 2 Laboratório de Geologia e Paleontologia, Instituto de Oceanografia, Universidade Federal do Rio Grande, Rio Grande, Rio Grande do Sul, Brazil; 3 New Mexico Museum of Natural History and Science, Albuquerque, NM, USA

**Keywords:** Pirambóia Formation, Ichnology, *Dicynodontipus*, *Chelichnus*, Paleoerg, Dicynodontia, Vertebrate tracks, South America, Permian–Triassic boundary

## Abstract

Tetrapod tracks in eolianites are widespread in the fossil record since the late Paleozoic. Among these ichnofaunas, the ichnogenus *Chelichnus* is the most representative of the Permian tetrapod ichnological record of eolian deposits of Europe, North America and South America, where the *Chelichnus* Ichnofacies often occurs. In this contribution, we describe five sets of tracks (one of which is preserved in cross-section), representing the first occurrence of *Dicynodontipus* and *Chelichnus* in the “Pirambóia Formation” of southern Brazil. This unit represents a humid desert in southwestern Pangea and its lower and upper contacts lead us to consider its age as Lopingian–Induan. The five sets of tracks studied were compared with several ichnotaxa and body fossils with appendicular elements preserved, allowing us to attribute these tracks to dicynodonts and other indeterminate therapsids. Even though the “Pirambóia Formation” track record is sparse and sub-optimally preserved, it is an important key to better understand the occupation of arid environments by tetrapods across the Permo–Triassic boundary.

## Introduction

Tetrapods experimented with their first incursions into desert environments during the Carboniferous Period and, since then, they have come to occupy almost all desert elements, such as dunes, interdunes and sand sheets (Krapovickas et al., 2016). Although the composition of the desert ichnofaunas has changed through the Phanerozoic (e.g., the replacement of the *Chelichnus* ichnocoenosis by the *Brasilichnium* ichnocoenosis after the Permo–Triassic boundary), the relative abundance of tetrapod-related ichnotaxa in such environments has always been low (Hunt & Lucas, 2007; Hunt & Lucas, 2016; Krapovickas et al., 2016). On the other hand, tetrapod tracks comprise the only fossil record of tetrapods in several eolian deposits across the world (Gilmore, 1926; Faul & Roberts, 1951; Leonardi, 1980; Lockley et al., 1995; Morales & Haubold, 1995; Dentzien-Dias, Schultz & Bertoni-Machado, 2008; Francischini et al., 2015), making the ichnotaxonomic and facies studies of such tetrapod tracks extremely important to understanding the evolution of biodiversity in and the occupation of arid ecosystems throughout geological time.

Among the late Paleozoic and early Mesozoic record, the main desert tetrapod ichnofaunas comes from the Permian eolianites of Scotland (Locharbriggs, Corncockle and Hopeman sandstones), Germany (Cornberg Sandstein), the western USA (Coconino, DeChelly, Lyons and Casper sandstones) and Argentina (Yacimiento Los Reyunos and Patquía formations) (Jardine, 1853; Lull, 1918; Gilmore, 1926; McKee, 1944; Schmidt, 1959; Cei & Gargiulo, 1977; Fichter, 1994; Lockley et al., 1995; Morales & Haubold, 1995; Krapovickas et al., 2010; Krapovickas et al., 2014). However, despite the terrestrial tetrapod faunal turnover and extinctions that marked the Guadalupian–Lopingian transition (Day et al., 2015a; Lucas, 2017) and the end-Permian biotic crisis (Benton & Twitchett, 2003; Retallack, Smith & Ward, 2003; Lucas, 2009), the Permian eolian tetrapod track record is dominated by *Chelichnus* tracks, which are morphologically constant during the entire Permian (McKeever & Haubold, 1996). This dominance is partially explained by the role of the preservation of tetrapod tracks in eolian sands, which add new non-morphological, substrate-controlled features to the original autopodium impression, referred to as extramorphological characters (Peabody, 1948; Haubold et al., 1995; Mancuso et al., 2016). Also, species that lived in arid *ergs* often present similar adaptations to walk on desert eolian substrates (such as short and broad digits, wider than long soles and palms, and the lack of a tail dragging on the ground). Therefore, the recurrence of the morphological and extramorphological features of the tetrapod tracks made on eolian sand substrates results in desert facies-controlled ichnofaunas, which are broadly known as the *Chelichnus* Ichnofacies (Lockley et al., 1995; Hunt & Lucas, 2007; Hunt & Lucas, 2016).

Several studies argued that the *Chelichnus* Ichnofacies is a depauperate association of tetrapod tracks in eolian deserts, being particularly less diverse than other contemporaneous ichnofaunas produced in different environments (Lockley et al., 1995; McKeever & Haubold, 1996; Hunt & Lucas, 2007; Hunt & Lucas, 2016). The *Chelichnus* Ichnofacies is dominated mainly by Chelichnopodidae tracks, being represented by *Chelichnus* Jardine, 1850 in the Paleozoic deposits, and *Brasilichnium* Leonardi, 1981 in the Mesozoic deposits, in addition to surface-made arthropod tracks—such as *Diplichnites*, *Hexapodichnus*, *Paleohelcura* and *Octopodichnus* (Brady, 1947; Leonardi, 1980; Braddy, 1995; Hunt & Lucas, 2007; Ekdale & Bromley, 2012; Hunt & Lucas, 2016).

**Figure 1:**
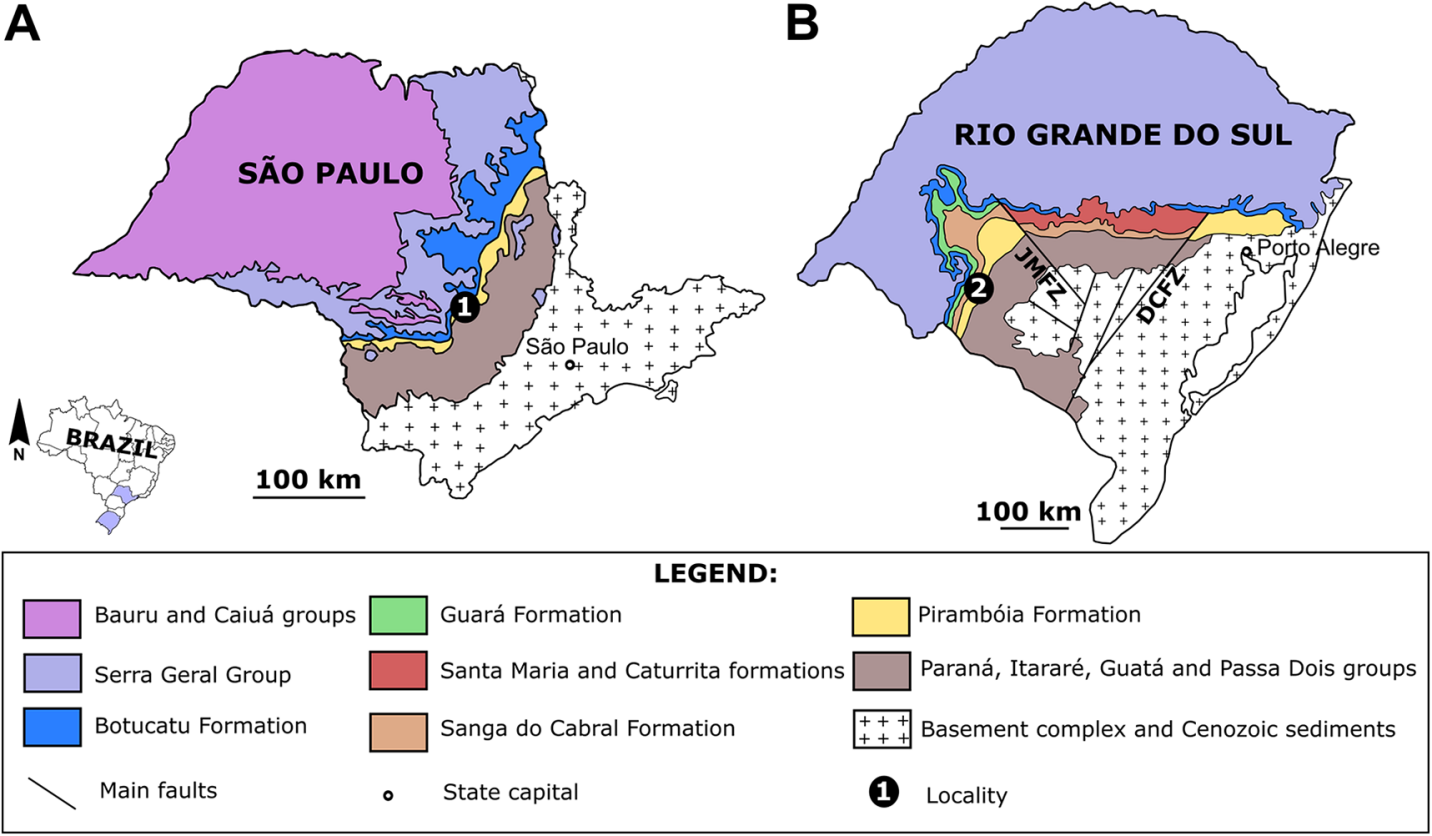
The Pirambóia Formation in São Paulo and Rio Grande do Sul states, Brazil. (A) São Paulo State. Locality 1: The type-locality in the Pirambóia district, Anhembi municipality. (B) Rio Grande do Sul State. Locality 2: The Ibicuí d’Armada locality, Santana do Livramento municipality. JMFZ, Jaguari-Mata Fault Zone; DCFZ, Dorsal de Canguçu Fault Zone. Modified from Scherer & Lavina (2005).

Here, we describe the first tetrapod tracks from the Lopingian–Induan eolian strata of southern Brazil, which are identified as *Dicynodontipus* isp. and *Chelichnus bucklandi*, besides other indeterminate trackways. These materials are the first tetrapod ichnocoenosis from an eolian environment in the late Paleozoic–early Mesozoic strata of Brazil, allowing the recognition of the *Chelichnus* Ichnofacies in the eolian dunes of the “Pirambóia Formation” from southwestern Rio Grande do Sul (Fig. 1). In addition, the ichnogenus *Dicynodontipus* is not often found in eolian deposits, making this record important in the understanding of the role of the extramorphological variations among tetrapod tracks.

## Geological Setting

### Stratigraphic setting

**Figure 2:**
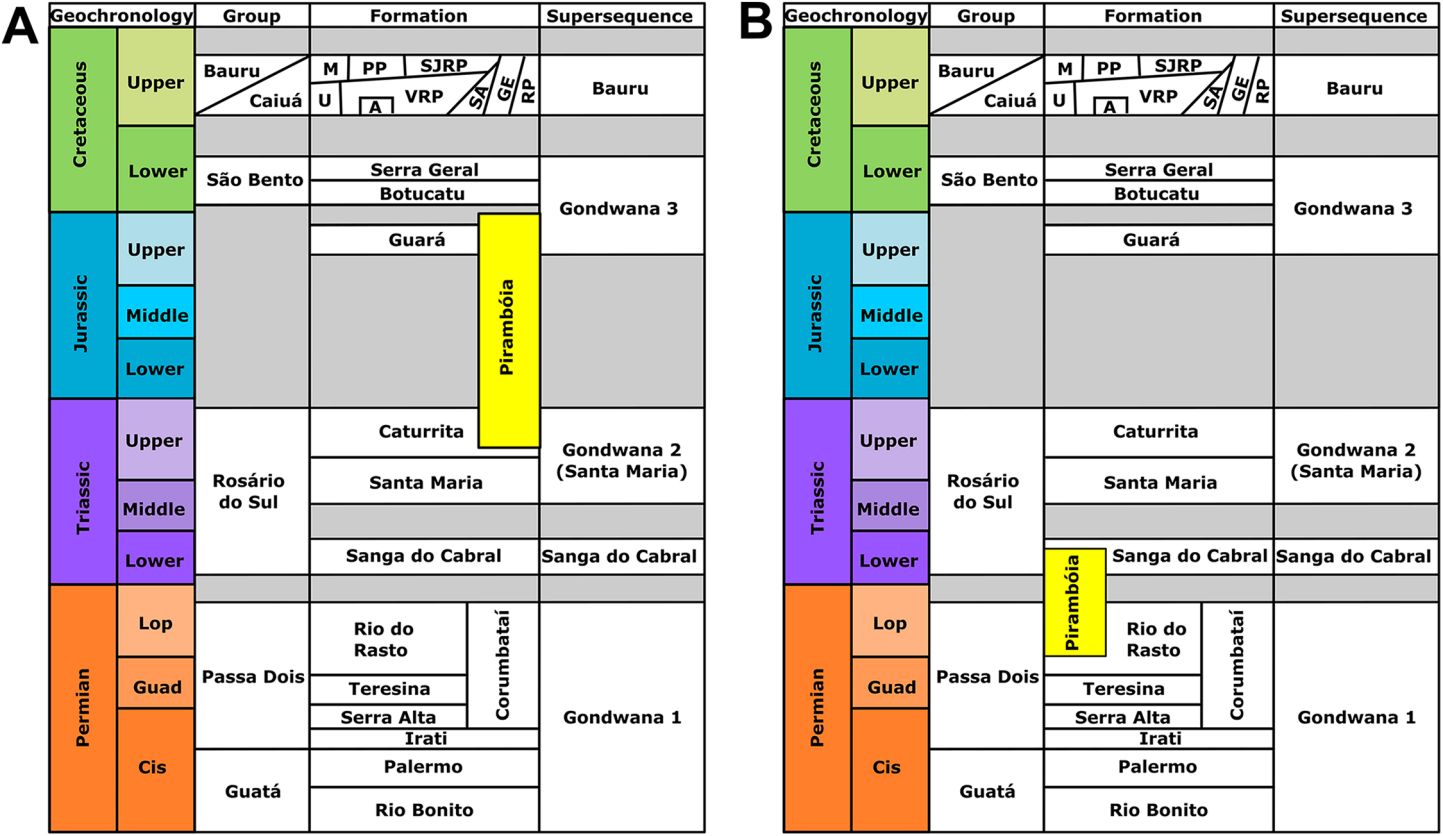
The disputed stratigraphic position of the Pirambóia Formation. (A) The Pirambóia Formation as partly chronocorrelated to the Caturrita and Guará formations (Triassic–Jurassic in age). This deposit occurs in the São Paulo State and in the eastern region of the Rio Grande do Sul State (east to the Dorsal de Caguçu Fault System). (B) The “Pirambóia Formation” that occurs in the western region of the Rio Grande do Sul State (west to the Jaguari-Mata Fault System). Note that the stratigraphic range of this unit is delimited by the Rio do Rasto (lower contact) and the Sanga do Cabral (upper contact) formations, providing a Lopingian–Induan age. Adapted from Soares, Soares & Holz (2008).

The Pirambóia Formation was proposed as a formal lithostratigraphic unit in the Pirambóia district, Anhembi municipality, central region of São Paulo State, southeastern Brazil (Soares, 1975). Its type section is located in the surroundings of the Marechal Rondon (SP-300) highway and the unit is stratigraphically positioned between the upper Permian Passa Dois Group and the Lower Cretaceous Botucatu Formation (Soares, 1975; Figs. 1 and 2). Its lithologic characteristics allow the informal division of the unit into two members: the lower, composed mainly of clayey sandstones, with plano-parallel and small cross-bedded stratifications; and the upper, composed of sandstones with medium scale planar cross-stratification, overlain by sandstones and mudstones with plano-parallel stratification (Soares, 1975). Later, some authors (see historical review in Lavina, Faccini & Ribeiro, 1993) described stratigraphic units with a similar lithology and stratigraphic position in Paraná, Santa Catarina and Rio Grande do Sul states, which led them to consider these units to be the same as the Pirambóia Formation of São Paulo State, despite the lack of continuous exposure along the eastern border of the Paraná Basin (Fig. 1; Lavina, Faccini & Ribeiro, 1993). Notwithstanding, the stratigraphic correlation of several units attributed to the Pirambóia Formation is controversial, because there is no consensus about its lateral extent (Fig. 2; Lavina, Faccini & Ribeiro, 1993; Soares, Soares & Holz, 2008). Hitherto, fossils were unknown at both the type-locality and across the studied region (except by those described herein). In this section, the stratigraphic relationships of the Pirambóia Formation will be discussed, focusing on the deposits assigned to it in the southwestern portion of Rio Grande do Sul State (Southern Brazil) where the tracks described in this work were discovered.

The occurrence of these clay, silt and clayey sandstone beds in São Paulo State was recognized for the first time in the reports of the Comissão Geográfica e Geológica do Estado de São Paulo (“Geographical and Geological Survey of the São Paulo State”), that named it the “Grês de Pirambóia,” located under the “Grês de Botucatu” (actually, the Botucatu Formation, composed mainly of eolian sandstones) layers (Pacheco, 1927; Washburne, 1930). According to Pacheco (1927), the “Grês de Pirambóia” was a sandy Triassic unit that crops out only in São Paulo State. Although the early reports did not characterize this unit stratigraphically, Sanford & Lange (1960) raised it to the formal category of formation.

Soares (1975) was the first to define the Pirambóia Formation based on a type section and to delimit its occurrence in São Paulo State (Fig. 1). Accordingly, the Pirambóia Formation differs from the sandstones of the (superposed) Botucatu Formation by being a predominantly fluvial facies association (Soares, 1975), although according to Caetano-Chang (1997) this is subordinate to the eolian facies. In São Paulo and Goiás states, the Pirambóia Formation overlies the Permian Passa Dois Group (i.e., Teresina, Rio do Rasto and Corumbataí formations), and its lower boundary is marked by a debrite level, informally named the “Porangaba Bed” (Matos, 1995; Matos & Coimbra, 1997). This bed was related to a tsunami deposit generated after the Araguainha impact event and a coherent population of detrital zircons was dated in 253.2 ± 3.0 Ma (Changhsingian, late Permian), suggesting that the overlying Pirambóia Formation is younger than this age (Tohver et al., 2018). However, there is no consensus about the nature (transitional or discordant) of this boundary in other regions of the Paraná Basin (Fúlfaro, Gama & Soares, 1980; Almeida & Melo, 1981; Lavina, 1992; Lavina, Faccini & Ribeiro, 1993; Faccini, 2000; Dias & Scherer, 2008).

In Rio Grande do Sul State, Lavina, Faccini & Ribeiro (1993) proposed that the lower beds of the Sanga do Cabral Formation (*sensu* Andreis, Bossi & Montardo, 1980) may be correlated to the Pirambóia Formation, as defined in São Paulo (Figs. 1 and 2). These beds, composed of an association of fine- to medium-grained sandstones with trough cross-bedded stratification, predominantly eolian in origin (but with subordinate lacustrine and fluvial levels), differ from the overlying succession (named the Sanga do Cabral Formation *strictu sensu*), composed of fluvial, lacustrine, deltaic and eolian mudstones and sandstones (Lavina, 1992; Lavina, Faccini & Ribeiro, 1993), with a tetrapod body fossil record (e.g., *Procolophon trigoniceps*) that indicates an Early Triassic age (Induan; Dias-da-Silva et al., 2017). On the other hand, the basal strata of the Pirambóia Formation in Rio Grande do Sul State contacts the top of the Rio do Rasto Formation, whose tetrapod (e.g., pareiasaurs, dinocephalians, anomodonts, among others), plant (e.g., the *Glossopteris* flora) and conchostracan records suggest a Guadalupian–Lopingian age (Holz et al., 2010; Dias-da-Silva, 2012). The exposures of the Pirambóia Formation are interrupted in the central region of Rio Grande do Sul State by two fault systems (the Jaguari-Mata Fault Zone, NW–SE, and the Dorsal de Canguçu Fault Zone, NE–SW), that restrict the occurrence of this unit to the southwestern and eastern regions of the State (Figs. 1 and 2) (Soares, Soares & Holz, 2008).

More recently, Soares, Soares & Holz (2008) recognized a conflict between the interpretations of the sandstone packages described by Lavina (1992) as Pirambóia Formation (that crops out west of the Jaguari-Mata Fault Zone) and those from the eastern region of Rio Grande do Sul (Fig. 2). According to these authors (Soares, Soares & Holz, 2008), the western package would have been deposited during the Permo–Triassic interval, based on its stratigraphic relationships with the lower and upper formations, the Rio do Rasto and the Sanga do Cabral formations, respectively (Lavina, Faccini & Ribeiro, 1993; Soares, Soares & Holz, 2008). On the other hand, the package that crops out in eastern Rio Grande do Sul (east of the Dorsal de Canguçu Fault Zone) should be chrono-correlated to the Upper Jurassic Guará Formation (Soares, Soares & Holz, 2008; Scherer & Lavina, 2005). Additionally, the “Porangaba Bed” does not occur in the Rio Grande do Sul State (Tohver et al., 2018), precluding further correlations with the deposits of the north region of the Paraná Basin. Therefore, to avoid conflict, we will refer to the eolianites of western Rio Grande do Sul as “Pirambóia Formation” (between quotes) henceforward.

Regarding the Pirambóia Formation fossil record, deposits bearing two associations described as belonging to the Santana Facies (lacustrine and flood-plain deposits) of the Botucatu Formation (Almeida, 1950; Souza, Sinelli & Gonçalves, 1971) were included in the Pirambóia Formation, in the definition proposed by Soares (1975). The fauna described by Almeida (1950) is composed of conchostracans (*Bairdestheria barbosai*, *Euestheria mendesi* and *Palaeolimnadia petrii*) and ostracods (*Candona*? sp., *Candonopsis pyriformis* and *Pachecoia rodriguesi*) from the mudstones of the Rio Claro municipality (central São Paulo State). According to this author, this fauna indicates a Triassic age (Almeida, 1950).

The second fossil assemblage, described by Souza, Sinelli & Gonçalves (1971) from the clayey rhythmites of Serrana municipality (northeastern São Paulo State), is composed of an abundant fauna of ostracods (*Cypridea oblonga*) and conchostracans (*Estheriella* sp., *E. ribeiropretensis*, *E. triangularis*, *Lioestheria elliptica* and *Pseudestheria* sp.), besides remains of the lycopsid plant *Lycopodiopsis derbyi*. Based on this, the age of this association is contradictory: while *L. derbyi* indicates a Permian age, *C. oblonga* suggests a Jurassic–Cretaceous age for those beds (Souza, Sinelli & Gonçalves, 1971). On the other hand, other than the trace fossils described here (see below), the “Pirambóia Formation” of western Rio Grande do Sul has no fossil record. Therefore, there is no consensus on the spatial and temporal definitions of the Pirambóia Formation, even in the original area in São Paulo State.

The material described in this work comes from the eolian sandstone package that crops out west of the Jaguari-Mata Fault Zone, being stratigraphically positioned between the Rio do Rasto and the Sanga do Cabral formations, corresponding, to the Pirambóia Formation *sensu* Lavina (1992). Accordingly, the tetrapod track-bearing eolian sandstones of the Santana do Livramento municipality were deposited during the interval late Lopingian–Induan (late Permian–Early Triassic). This inferred age is based on the stratigraphic position of this package between the Rio do Rasto and the Sanga do Cabral formations.

### Geology and meaning of the “Pirambóia Formation” in southwestern Rio Grande do Sul

Despite all the stratigraphic contradictions described above, it is clear that the fluvio-eolian deposits from southwestern Rio Grande do Sul State represent a humid eolian system deposited in the interval Guadalupian–Induan (Dias & Scherer, 2008). The depositional age of this unit is based on its lower and upper unconformable contacts with the Rio do Rasto Formation (Guadalupian–Lopingian) and the Sanga do Cabral Formation (Induan) (Dias & Scherer, 2008; Soares, Soares & Holz, 2008; Rodrigues, 2014; Soares, Soares & Bettú, 2014).

In southwestern Rio Grande do Sul State, the “Pirambóia Formation” is composed of 10 lithofacies that indicate deposition under eolian settings with braided and ephemeral fluvial channels (Rodrigues, 2014). A drying-upward trend is proposed based mainly on the high frequency of sandy sheets and interdune deposits in the lower half and the predominance of eolian dunes in the upper half of the “Pirambóia Formation” (Rodrigues, 2014) and the increase in thickness of the dune deposits upward through the entire unit (Dias & Scherer, 2008).

Biogenic structures were observed in at least three lithofacies: eolian sandy sheets, dry and wet eolian interdunes and eolian dunes (Rodrigues, 2014). Even though these trace fossils were not properly described, Rodrigues (2014) was able to recognize the *Scoyenia* Ichnofacies (i.e., an ichnofacies characterized mainly by the co-occurrence of vertebrate and invertebrate mobile deposit feeding traces and locomotion tracks and trails, besides dwelling burrows and rhizoliths; e.g., McEachern et al., 2012 and references therein) in the interdune deposits.

## Materials and Methods

**Figure 3:**
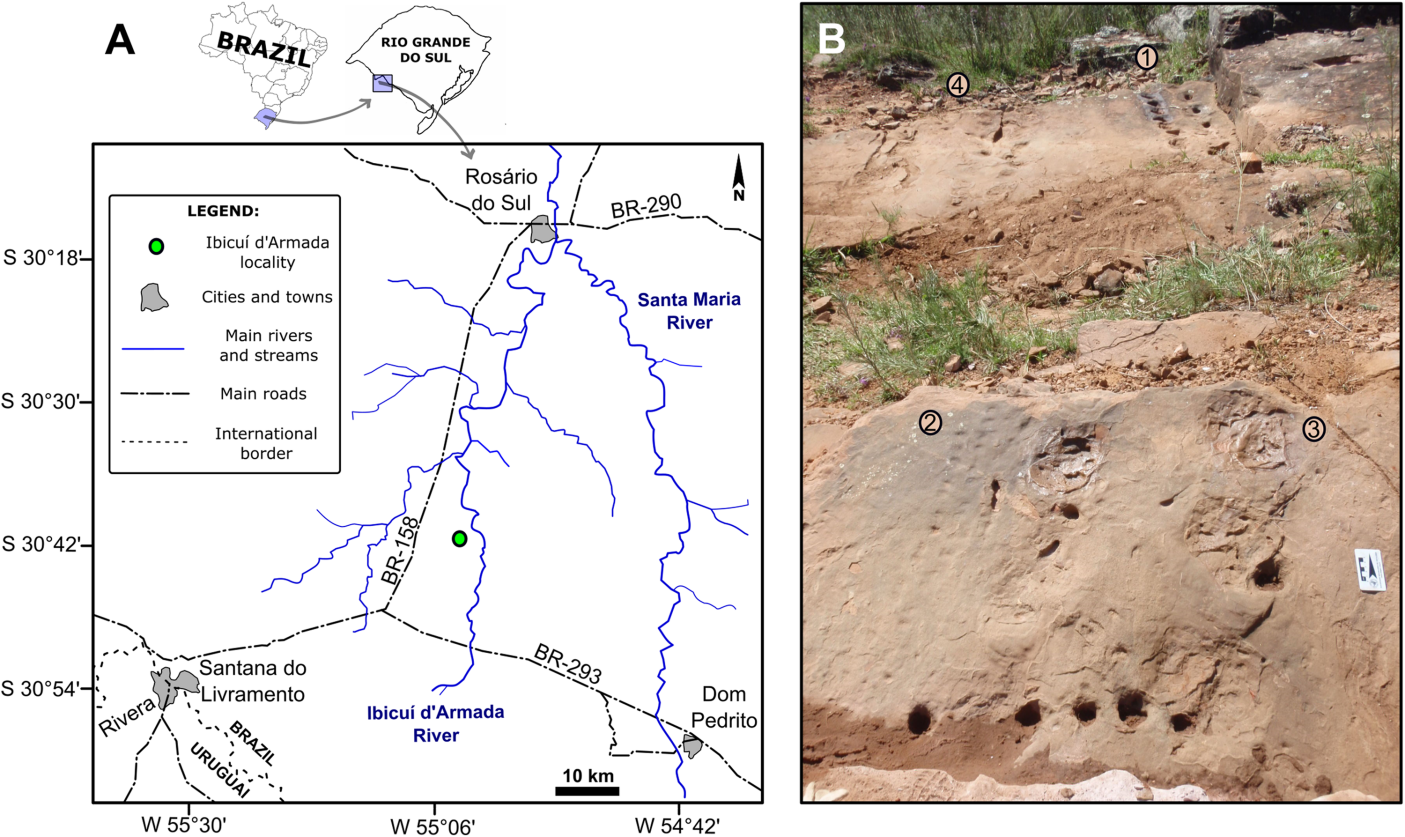
The Ibicuí d’Armada locality, southwest Rio Grande do Sul State, southern Brazil. (A) Geographic locality of the outcrop. (B) General view of the outcrop. 1: SLIA-1, 2: SLIA-2, 3: SLIA-3, 4: SLIA-4. Scale: 5 cm.

The track-bearing outcrop studied in this contribution (Coordinates: UTM 21J 0687503/6600663; Fig. 3) is located in the Santana do Livramento municipality, in the southwestern region of Rio Grande do Sul State, southern Brazil (Fig. 3A). The outcrop is an exposure on the right side of an unnamed secondary road, west of the Ibicuí d’Armada River, which gave its name to the region. In the entire region it is possible to see eolian deposits cropping out, though the fossil tracks are found *in situ* only in one small area of 10 m^2^ (Fig. 3B). Permit for field work in this area was provided by the Departamento Nacional de Produção Mineral (Process Number: 000.820/2015).

Apparently, a single eolian dune deposit is exposed in the Ibicuí d’Armada outcrop, reaching nearly 1.5 m of height. This deposit is composed of a set of inverse graded strata with dip angles of 20° and 32°. The eolian paleoflow azimuths vary between 150° and 230°, with a mean of 187°. All these data were collected *in situ*. Beside the tracks described below, a tetrapod burrow also occurs in the same strata (Fig. S1 in the Supplemental Information File).

A total of five trackways (four complete trackways in plan view and one in cross-section) was discovered in the Ibicuí d’Armada outcrop. All the trackways were represented by four letters (SLIA), the first two referring to the municipal district (SL, Santana do Livramento) and the last two to the locality (IA, Ibicuí d’Armada). Arabic numbers differentiate the trackways among themselves (SLIA-1 to SLIA-5). The Roman numbers following the trackway abbreviation represents each footprint in the order of the successive set in the trackway. The trackways SLIA-1 and SLIA-4 occur in the same eolian stratum with a 20° inclination, while SLIA-2, SLIA-3 and SLIA-5 occur in another, with a 32° inclination.

The trackways were photographed *in situ* and subsequently replicated as silicon rubber casts that are housed in the Laboratório de Paleontologia de Vertebrados of the Universidade Federal do Rio Grande do Sul (UFRGS), in Porto Alegre (Brazil), under the collection numbers UFRGS-PV-0391-P (mold of the SLIA-1 tracks) and UFRGS-PV-0392-P (mold of the surface that contains the SLIA-2 and SLIA-3 tracks). Additionally, trackway SLIA-2 was collected and deposited in UFRGS under the number UFRGS-PV-0601-P. The quantitative and qualitative parameters of each footprint and the whole trackways were obtained based on the methodology proposed by Leonardi (1987). The morphology (i.e., number and shape of digits, autopodium axis, position of the autopodium) and measurements (i.e., width and length of each footprint, the divarication of the digits, length of the pace, oblique pace and stride, pace angulation, distance between *manus* and *pes* and the divarication of the *manus* from the midline) were obtained *in situ* using a measure tape and a caliper and confirmed using the free software ImageJ®. The trackway SLIA-5 (collected and deposited in UFRGS under the number UFRGS-PV-0602-P) is preserved in cross-section and its study follows the criteria proposed by Loope (1986). The gleno-acetabular distance (i.e., the distance between the center of the glenoid cavity and the center of the acetabular cavity) was estimated based on the measurement of the distance between the intersections with the midline of the line of the hands and of the line of the feet with both these lines being more or less subparallel (Leonardi, 1987).

**Figure 4:**
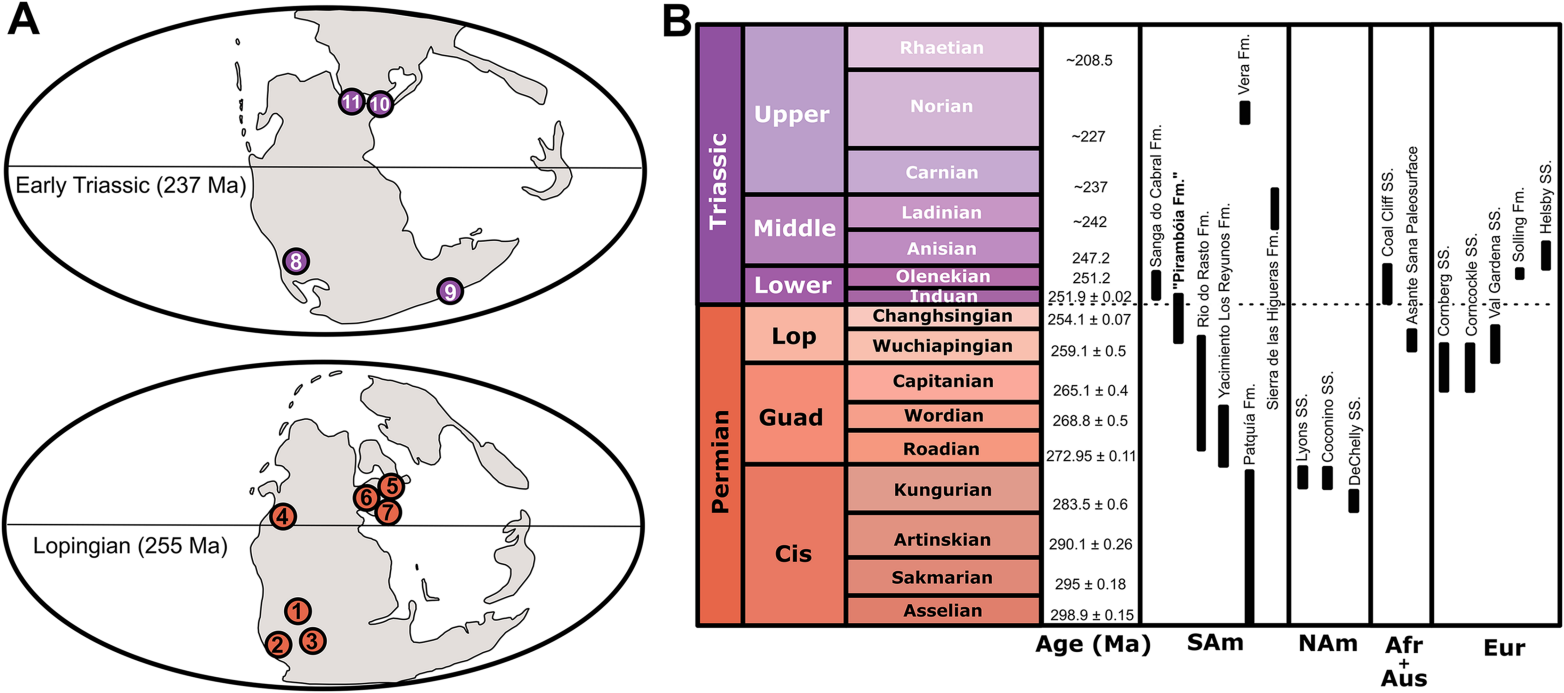
Paleogeographic and chronostratigraphic occurrence of the main units mentioned in the text. (A) Paleogeographic maps showing the main late Permian–Early Triassic *Dicynodontipus*- and *Chelichnus*-bearing localities: 1. Brazil (“Pirambóia Formation”), 2. Argentina (Yacimiento los Reyunos and Patquía formations), 3. South Africa (Asante Sana Paleosurface, Oudeberg Member of the Balfour Formation), 4. Western United States (Coconino, DeChelly and Lyons sandstones), 5. Germany (Cornberg Sandstein), 6. Scotland (Corncockle Sandstone), 7. Italy (Val Gardena Sandstone), 8. Argentina (Vera and Sierra de las Higueras formations), 9. Australia (Coal Cliff Sandstone), 10. Germany (Solling Formation), 11. England (Helsby Sandstone). (B) Chronostratigraphic position of the same units. Ages were taken from: Bonaparte (1966), Haubold (1971a, 1971b), Retallack (1996), De Klerk (2002), King et al. (2005), Lucas & Hunt (2006), Melchor & de Valais (2006), Krapovickas et al. (2010), Ogg (2012), Krapovickas et al. (2014), Dias-da-Silva et al. (2017), Marchetti, Voigt & Klein (2017) and Francischini et al. (2018). See text for further information. Maps modified from Scotese (2002).

A total of three approaches were used to determine the identities of the probable trackmakers. Firstly, the morphology and measurements were compared with several mid- to large-sized ichnotaxa found in Permian–Triassic deposits (Fig. 4). The main comparisons were made with the quadrupedal ichnotaxa recorded in eolian facies, such as *Chelichnus* Jardine, 1850 (including the type material proposed by Lull, 1918 and Gilmore, 1926 as “*Laoporus*,” “*Agostopus*,” “*Allopus*,” “*Baropezia*,” “*Barypodus*,” “*Dolichopodus*,” “*Nanopus*” and “*Palaeopus*,” all of them considered junior synonyms of *Chelichnus* by McKeever & Haubold, 1996); *Navahopus* Baird, 1980; and *Brasilichnium* Leonardi, 1981. However, in order to better understand the role of extramorphological variation of the Ibicuí d’Armada tracks, they were also compared with Permian–Cretaceous ichnotaxa produced in fluvio-lacustrine and volcaniclastic facies, such as *Ameghinichnus* Casamiquela, 1961; *Brontopus* Heyler & Lessertisseur, 1963; *Catocapes* Mateus et al., 2017; *Dicynodontipus* Rühle von Lilienstern, 1944 (including “*Calibarichnus*” and “*Gallegosichnus*”; Casamiquela, 1964); *Pachypes* Leonardi et al., 1975 (including “*Sukhonopus*”; Gubin et al., 2003); and *Therapsipus* Hunt et al., 1993.

In addition, the anatomy of the *manus* and *pedes* of Permo–Triassic tetrapods was analyzed, mainly in those taxa whose complete phalangeal formula was preserved, based on the available published data (see the bibliography). Lastly, the faunal composition of deposits of the same age (Lopingian–Induan) as that inferred for the “Pirambóia Formation” was considered.

## Results

### Systematic Paleoichnology

***Dicynodontipus*Rühle von Lilienstern, 1944**

**Type ichnospecies.** *Dicynodontipus hildburghausensis* Rühle von Lilienstern, 1944.

**Diagnosis.** Relatively narrow trackways, pace angulation at normal gait at least 100°, at higher pace angulation *manus* impressions can be overstepped, only at lower pace angulation *manus* impressions are positioned at short distance anterior to the feet. *Manus* and *pes* showing the same shape, plantigrade, pentadactyl; short, anteriorly orientated digits, digit IV the longest, digit V slightly laterally and posteriorly shifted (modified from Melchor & de Valais, 2006).

**Age and occurrence.** Permian–Triassic strata of Germany (Solling Formation, Buntsandstein of Thuringia), Italy (Val Gardena Sandstone of the Dolomites region), England (Helsby Sandstone of Cheshire), South Africa (Oudeberg Member of the Balfour Formation, Beaufort Group, Karoo Basin), Australia (Coal Cliff Sandstone of the Sidney Basin), Argentina (Vera Formation of the Los Menucos Depocentre; Sierra de las Higueras Formation of the Las Higueras-Santa Clara Basin; and Cerro de las Cabras Formation of the Cuyo Basin) and Brazil (Rio do Rasto and “Pirambóia” formations of the Paraná Basin) (Fig. 4; Table 1).

**Table 1:** Summary of the main ichnotaxonomic changes of the materials assigned to *Dicynodontipus*.

Original description	Age and locality	Other interpretations
*Dicynodontipus hildburghausensis* (Rühle von Lilienstern, 1944)*	Lower Triassic of Thuringia, Germany	*Chelichnus geinitzi* (Haubold, 1965) *Dicynodontipus geinitzi* (Haubold, 1971a, 1971b)
*Chirotherium geinitzi* (Hornstein, 1876)	Lower Triassic of Thuringia, Germany	*Chelichnus geinitzi* (Haubold, 1965; Kuhn, 1963)
		*Dicynodontipus geinitzi* (Haubold, 1971a, 1971b)
*Onkichnium beasleyi* (Kuhn, 1963)	Lower Triassic of Thuringia, Germany	*Dicynodontipus geinitzi* (Haubold, 1971a, 1971b)
*Agostropus falcatus* (Rühle von Lilienstern, 1939)	Lower Triassic of Thuringia, Germany	*Dicynodontipus geinitzi* (Haubold, 1971a, 1971b)
*Dicynodontipus geinitzi* (Conti et al., 1977)	Lopingian of Trentino-Alto Ádige, Italy	*Dicynodontipus* isp. (Avanzini et al., 2001; Avanzini & Tomasoni, 2004; Marchetti, Voigt & Klein, 2017) *Dicynodontipus geinitzi* (Avanzini, Bernardi & Nicosia, 2011; Bernardi et al., 2017)
*Dicynodontipus icelsi* (De Klerk, 2002)	Lopingian of the Eastern Cape, South Africa	cf. *Dolomitipes* isp. (Marchetti, Voigt & Klein, 2017)
*Dicynodontipus bellambiensis* (Retallack, 1996)	Lower Triassic of New South Wales, Australia	–
*Calibarichnus ayesterani* (Casamiquela, 1964)	Upper Triassic of Río Negro, Argentina	*Dicynodontipus* isp. (Melchor & de Valais, 2006)
*Gallegosichnus garridoi* (Casamiquela, 1964)	Upper Triassic of Río Negro, Argentina	*Dicynodontipus* isp. (Melchor & de Valais, 2006)
*Palaciosichnus zetti* (Casamiquela, 1964)	Upper Triassic of Río Negro, Argentina	*Dicynodontipus* isp. (Melchor & de Valais, 2006)
*Stipanicichnus bonnetti* (Casamiquela, 1975)	Upper Triassic of Río Negro, Argentina	*Dicynodontipus* isp. (Melchor & de Valais, 2006)
cf. *Dicynodontipus* (Leonardi, 1994)	Middle Triassic of Mendoza, Argentina	–
cf. *Dicynodontipus* (Leonardi, Sedor & Costa, 2002)	Guadalupian–Lopingian of Paraná, Brazil	*Dicynodontipus* isp. (Silva, Sedor & Monteiro-Filho, 2012) *Dicynodontipus penugnu* (Silva, Sedor & Fernandes, 2012) Non-*Dicynodontipus* (Marchetti, Voigt & Klein, 2017)
*Dicynodontipus protherioides* (Silva et al., 2008)	Upper Triassic of Rio Grande do Sul, Brazil	*Procolophonichnium* isp. (Klein, Lucas & Voigt, 2015)

**Note:**

The type material is indicated by the asterisk.

**Remarks.** The material that has been assigned to *Dicynodontipus* is highly variable in morphology and has a puzzling ichnotaxonomic history (Table 1). Rühle von Lilienstern (1944) erected this ichnogenus based on tracks from the Buntsandstein of Hildburghausen (Thuringia, Germany), coining the ichnospecies *D. hildburghausensis*. The material previously described by Hornstein (1876) as *Chirotherium geinitzi* was lately classified within the ichnogenus *Chelichnus* by Haubold (1965), proposing the new combination *C. geinitzi*. Some years later, Haubold (1971a, 1971b) reinterpreted both materials as *D. geinitzi*. Since then, several authors (e.g., Conti et al., 1977; Retallack, 1996; Melchor & de Valais, 2006; Silva et al., 2008; Silva, Sedor & Fernandes, 2012; Marchetti, Voigt & Klein, 2017, among others) have followed Haubold’s (1971a, 1971b) assignment, using *D. geinitzi* as the type-ichnospecies of *Dicynodontipus.* However, this is contrary to the Paragraph 61.1.3. of the Article 61 of the International Code of Zoological Nomenclature (ICZN, 1999), which claims that “the name-bearing type of any nominal taxon, once fixed in conformity with the provisions of the Code, is not subject to change.” Therefore, *D. hildburghausensis* must be considered the type-material of the ichnogenus *Dicynodontipus*, even if the name *D. geinitzi* is considered a senior synonym. Table 1 summarizes the main historical changes in the ichnotaxonomic interpretation of the materials attributed to *Dicynodontipus.*

Nevertheless, other materials from Argentina, Australia, Brazil, England, Italy and South Africa have been described since then (Fig. 4; Table 1). The specimens from the Lopingian Val Gardena Sandstone (northern Italy) were originally interpreted as *D. geinitzi* (Conti et al., 1977), but recently reinterpreted as *Dicynodontipus* isp. (Marchetti, Voigt & Klein, 2017). Melchor & de Valais (2006) also recognized the presence of four different ichnospecies of *Dicynodontipus* in the Upper Triassic Vera Formation of Río Negro, Argentina, all of them originally described by Casamiquela (1964, 1975) as distinct, endemic ichnogenera. In addition, Leonardi (1994) reported the presence of cf. *Dicynodontipus* in the Sierra de las Higueras Formation of Mendoza, also in Argentina. Although the age of this unit is not well-known, Bonaparte (1966) proposed a Ladinian age for these tracks.

Leonardi, Sedor & Costa (2002) described the presence of *Dicynodontipus* isp. in the Guadalupian–Lopingian Morro Pelado Member of the Rio do Rasto Formation from the Paraná State, Brazil. This record was later revisited by Silva, Sedor & Fernandes (2012), who proposed a new ichnospecies: *D. penugnu*. Silva et al. (2008) described *D. protherioides* from the Upper Triassic deposits of the Alemoa Member of the Santa Maria Formation (*Hyperodapedon* Assemblage Zone; Candelária Sequence) of the Rio Grande do Sul State, in southern Brazil. But these materials were reinterpreted by Klein, Lucas & Voigt (2015) as belonging to *Procolophonichnium.*

New ichnospecies were also described from the Lower Triassic of Australia (*D. bellambiensis*; Retallack, 1996) and the Lopingian of South Africa (*D. icelsi*; De Klerk, 2002). The later was reinterpreted by Marchetti, Voigt & Klein (2017) as belonging the ichnogenus *Dolomitipes*. Klein & Niedźwiedzki (2012) reported the presence of tracks similar to *Dicynodontipus* in the Olenekian Wióry Formation of the Holy Cross Mountain of southern Poland, but the incompleteness and suboptimal preservation do not allowed a definitive assignment.

***Dicynodontipus* isp.**

**Referred material.** The trackway SLIA-1, a set of 14 consecutive footprints, and the respective mold (UFRGS-PV-0391-P).

**Horizon and locality.** Ibicuí d’Armada locality (21J 0687503/6600663), Santana do Livramento municipality, southwestern region of Rio Grande do Sul State, southern Brazil; “Pirambóia Formation,” Lopingian–Induan of the Paraná Basin.

**Figure 5:**
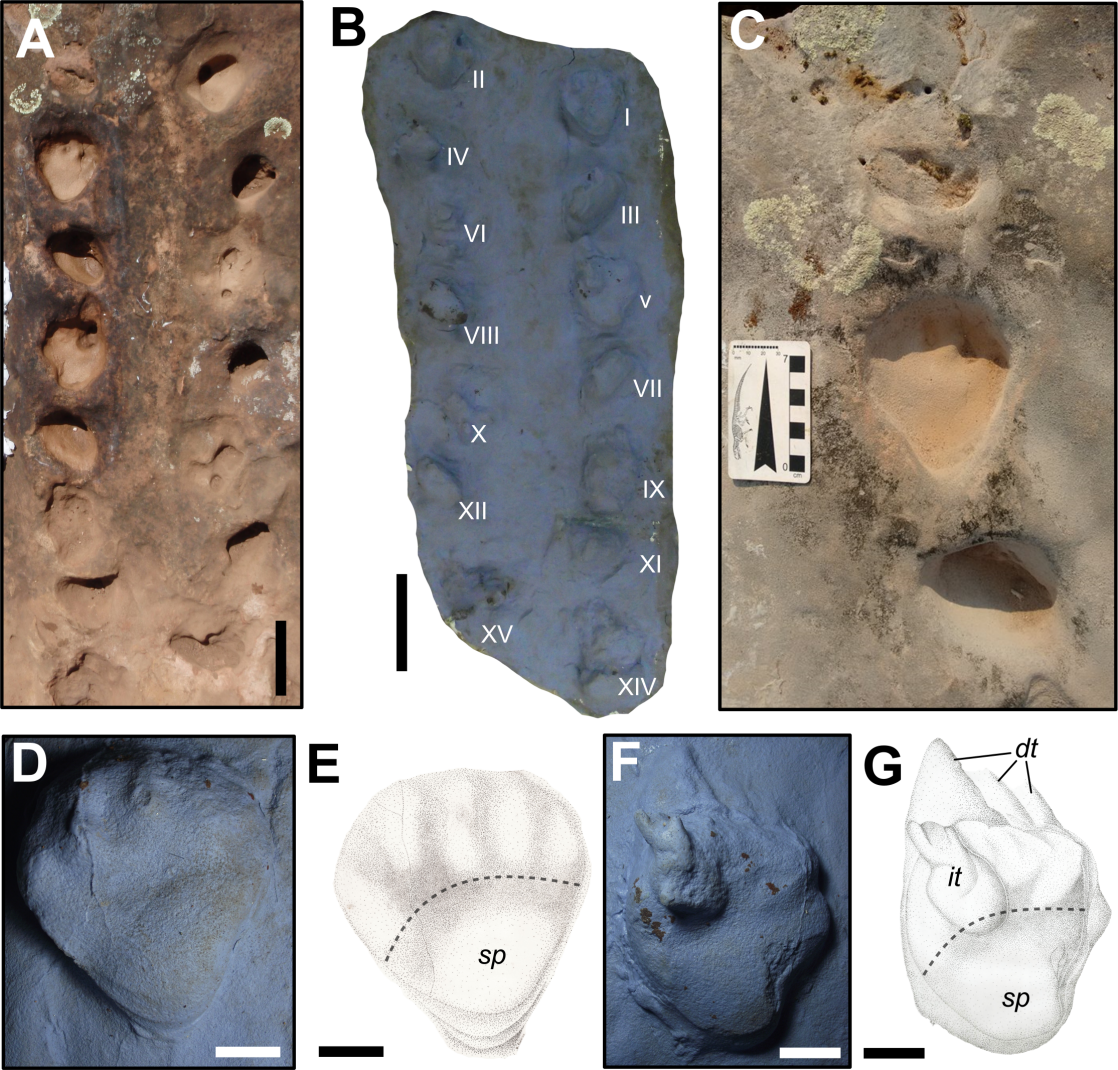
*Dicynodontipus* isp. from the “Pirambóia Formation,” Brazil. (A) General view of the trackway SLIA-1. (B) Silicon rubber mold of SLIA-1 (UFRGS-PV-0391-P). Roman numbers I–XV indicate each footprint. (C) Detail of the track SLIA-1-I (*pes*) and the relative *manus* tracks placed anteriorly and posteriorly. (D) Detail of the mold of SLIA-1-I (*pes*). (E) Schematic drawing of the preceding image. (F) Detail of the mold of SLIA-1-V (*pes*). (G) Schematic drawing of the preceding image. Dashed lines in (E) and (G) indicate the approximate location of the metapodial-phalangeal line. Abbreviations: dt, digital drag traces; it, possible invertebrate trace; sp, solar pad. Scales: 15 cm (A–B), 7 cm (C), 3 cm (D–G). Image credit: the authors (except for the drawings presented in (E) and (G): Sheron Medeiros).

**Description:** The SLIA-1 trackway consists of a set of 14 footprints (eight *pes* and six *manus* impressions) preserved as concave epireliefs and produced by a quadrupedal animal (Fig. 5). No tail- or body-drag traces were observed in association with the set of footprints. Claw-drag traces are not seen in both *manus* and *pedes*, except for the pedal track SLIA-1-V (Figs. 5F–5G). The mean internal and external trackway widths are 155 and 328.3 mm, respectively.

The *manus* imprints are pentadactyl, mesaxonic (i.e., the main digit is the central one; Leonardi, 1987) and semi-palmigrade to digitigrade (i.e., the tracks are formed only by the impressions of the anterior portion of the *manus* or only by the digits; Leonardi, 1987), with a straight proximal end of the palm. The mean sizes of the *manus* prints are 47.5 mm length and 72.33 mm width, with a width/length ratio of about 1.52 (Table S1 in the Supplemental Information File). The *manus* prints are deeper than the pedal ones, showing a mean depth of about 46 mm (Table S1 in the Supplemental Information File). The mean values of the manual oblique pace length, pace angulation and stride length are about 274.2 mm, 57.5° and 261.2 mm, respectively (Table S2 in the Supplemental Information File). The *manus* impressions show a negative (inward) divarication from the midline of about 30° (Table S1 in the Supplemental Information File) and are located about 165 mm from the associated *pes* print (Table S2 in the Supplemental Information File).

The *pes* prints are plantigrade (i.e., formed by the impression of the complete autopodium; Leonardi, 1987), nearly mesaxonic and also pentadactyl (Figs. 5C–5G). The heel is elongated, with a V-shaped sole in the proximal end, giving a subtriangular shape to the entire footprint. The *pedes* are directed forward, being sub-parallel to the midline. The mean sizes of the *pes* tracks are about 77.83 mm length and 86 mm width, with a width/length ratio about 1.10. (Table S1 in the Supplemental Information File). The mean of the pedal oblique pace length, pace angulation, stride length and depth are about 262.8 mm, 64.6°, 280.8 mm and 38.87 mm, respectively (Table S2 in the Supplemental Information File). The DPIA-1-I *pes* track has a well preserved sole pad, which covers almost the entire heel (Figs. 5D–5E). The proximo-lateral zone of the sole pad is very marked and represents the deepest part of the track. Also, at least one nearly round phalangeal pad can be recognized in each pedal digit of DPIA-1-I.

The gleno-acetabular distance of the trackmaker is estimated as 408.8 or 300.5 mm, respectively, considering alternate walk and amble gaits.

**Remarks.** As pointed out by McKeever & Haubold (1996), a digitigrade stance is not inferred from the Permian tetrapod body fossil record. According to these authors, the manual digitigrady present in some *Chelichnus* tracks (making reference to those from Scotland) is due to their preservation. Actually, it can be a variation related to the gait of a palmigrade producer, especially when made upslope or downslope in eolian sediments. Once the SLIA-1 travel direction is upslope, we use the same argument to explain the digitigrady of its *manus* tracks.

**Chelichnopodidae Lockley, 2011**
***Chelichnus*Jardine, 1850**

**Type ichnospecies.** *Chelichnus duncani* (*sensu* Owen, 1842) Jardine, 1850.

**Revised diagnosis.** Tetrapod trackways with mammal-like reptile (theromorph) characteristics; complete *manus* and *pes* impressions rounded and of nearly equal size; *manus* impressions usually slightly smaller in size and, apparently, more digitigrade in style; *pes* impression size ranges from approximately 10 mm up to approximately 200 mm in length. Complete *manus* and *pes* impressions show round pads with up to five short digits, although usually only three to four digits are found impressed; first four digits directed anteriorly and display low degree of divarication with fifth digit situated markedly postero-laterally; digits usually somewhat separated from sole. Normal trackway pattern shows *pes* pace angulation of up to 90°, with *manus* and *pes* impressed close together, or with slight overlap of *pes* on *manus* (after McKeever & Haubold, 1996).

**Age and occurrence.** Permian strata of Scotland (Corncockle Sandstone Formation of the Lochmaben Basin; Locharbriggs Sandstone Formation of the Dumfries Basin; and Hopeman Sandstone Formation of the Elgin area), Germany (Cornberger Sandstein of Hessen), southwestern USA (Coconino Sandstone, Arizona; DeChelly Sandstone, Arizona; Lyons Sandstone, Colorado; Cedar Mesa Sandstone, Utah; and Casper Sandstone, Wyoming and Colorado), Argentina (Yacimiento Los Reyunos Formation of the San Rafael Block and Patquía Formation of the Paganzo Basin) and Brazil (“Pirambóia Formation” of the Paraná Basin) (Fig. 4; Table 2).

**Table 2:** Summary of the main ichnotaxonomic changes of the materials assigned to *Chelichnus*.

Original description	Other interpretations
*Agostopus matheri* (Gilmore, 1926), *?Amblyopus* (Schmidt, 1959), *Baropezia eakini* (Gilmore, 1926), *Barypodus gravis* (Schmidt, 1959), *Barypodus metzeri* (Gilmore, 1927), *Barypodus mildei* (Schmidt, 1959), *Barypodus tridactylus* (Gilmore, 1927), *Chelichnus ambiguus* (Jardine, 1853), *Chelichnus locharbriggsensis* (McKeever, 1994), *Chelichnus plagiostopus* (Jardine, 1853), *Chelichnus ?tripodizon* (Schmidt, 1959), *Harpagichnus acutum* (Schmidt, 1959), *Herpetichnus loxodactylus* (Dudgeon, 1878), *Herpetichnus sauroplesius* (Jardine, 1850), *Laoporus noblei* (Lull, 1918), *Nanopus maximus* (Gilmore, 1927), *Palaeopus regularis* (Gilmore, 1926), *Palmichnus resinum* (Schmidt, 1959), *Phalangichnus alternans* (Schmidt, 1959), *Phalangichnus simulans* (Schmidt, 1959), *Testudo duncani* (Owen, 1842)*	*Chelichnus duncani* (McKeever & Haubold, 1996)
*Batrichnis lyelli* (Jardine, 1853), *Batrichnis stricklandi* (Harkness, 1851), *Cardiodactylum permicum* (Delair, 1966), *Chelaspodus jardini* (Harkness, 1851), *Chelichnus obliquus* (Harkness, 1851), *Chelichnus plancus* (Harkness, 1851), *Chelichnus pricei* (Delair, 1966), *Dolichopodis tetradactylus* (Gilmore, 1926), *Herpetichnus bucklandi* (Jardine, 1850), *Labyrinthodon lyelli* (Harkness, 1851), *Laoporus noblei* (Lull, 1918), *Laoporus schucherti* (Lull, 1918), *Nanopus merriami* (Gilmore, 1926), *Prochirotherium truckelli* (Delair, 1966), *Saurichnis acutus* (Harkness, 1851)	*Chelichnus bucklandi* (McKeever & Haubold, 1996)
*Amblyopus pachypodus* (Gilmore, 1927), *Barypodus palmatus* (Gilmore, 1926), *Chelichnus megacheirus* (Huxley, 1877), *Herpetichnus robustus* (Delair, 1966)	*Chelichnus gigas* (McKeever & Haubold, 1996)
*Allopus arizonae* (Gilmore, 1926)	*Chelichnus titan* (McKeever & Haubold, 1996)
*Chelichnus incurvus* (Gand, Demathieu & Ballestra, 1995)	–
*Chelichnus lutevanus* (Ellenberger, 1984)	–
*Chelichnus tazelwürmi* (Ceoloni et al., 1988)	*Contiichnus tazelwurmi* (Citton et al., 2017), *Contiichnus tazelwurmi* (Bernardi et al., 2017), *Procolophonichnium tirolensis* (Marchetti, Voigt & Klein, 2017)
Indeterminate tracks (Andreis & Carvalho, 2001)	*Tridactylichnium* isp. (Silva & Fernandes, 2004; Silva & Fernandes, 2005), *Chelichnus* isp. (Silva, Sedor & Fernandes, 2012), Non-*Chelichnus* (This paper)

**Note:**

The type material is indicated by the asterisk.

**Remarks.** Despite the proposition of McKeever & Haubold (1996) that the ichnogenus *Chelichnus* is restricted to the late Permian, and it should not be expanded to include trackways from older or younger strata, important material of *Chelichnus* has been described from the Coconino Sandstone (USA) and the Yacimiento Los Reyunos Formation (Argentina), both Cisuralian in age (Lull, 1918; Gilmore, 1926; Cei & Gargiulo, 1977; Krapovickas et al., 2014).

As in *Dicynodontipus, Chelichnus* encompasses a wide range of morphological variation in tracks made in eolian deposits. The ichnogenus was erected by Jardine (1850) in order to reallocate the tracks described by Owen (1842) as *Testudo duncani*. Several other similar ichnotaxa found in eolian deposits of Scotland, Germany, and the USA were erected (Table 2; e.g., Harkness, 1850; Harkness, 1851; Jardine, 1853; Huxley, 1877; Dudgeon, 1878; Lull, 1918; Gilmore, 1926, 1927; Schmidt, 1959; Delair, 1966; McKeever, 1994), but they were reassigned to *Chelichnus* by McKeever & Haubold (1996). In addition, besides the four *Chelichnus* ichnospecies recognized by McKeever & Haubold (1996) (i.e., *C. bucklandi, C. duncani, C. gigas* and *C. titan*), three others were also erected: *C. incurvus* Gand, Demathieu & Ballestra (1995), *C. lutevanus* Ellenberger (1984) and *C. tazelwürmi* Ceoloni et al. (1988). The latter was recently re-evaluated (Citton et al., 2017) and now it belongs to the ichnogenus *Contiichnus*, but the taxonomic meaning of the two former ichnospecies is still problematic.

Andreis & Carvalho (2001) described about 82 isolated tracks from the Pau Preto Quarry at the Taguaí municipality (São Paulo State), where the Guadalupian–Lopingian Corumbataí Formation crops out. According to the authors, these tracks are tridactyl and, based on the age of this unit (considered Lopingian–Early Triassic at that time), they were attributed to archosaurs. These tracks were not collected and were subsequently destroyed. Silva & Fernandes (2004, 2005) attributed preliminarly these tracks to *Tridactylichnium* isp. (a *nomen dubium* according to Marchetti, Belvedere & Mietto, 2017) and, more recently, Silva, Sedor & Fernandes (2012) redescribed these tracks, based on digitally enhanced versions of the original images of Andreis & Carvalho (2001), and attributed them to *Chelichnus* isp. However, there are some discrepancies between the poorly preserved morphology of the tracks and the interpretive drawing of Silva, Sedor & Fernandes (2012), such as the estimation of the digit count and the outline of each imprint. Therefore, we do not consider valid the ichnotaxonomy proposed for the Corumbataí tracks (Silva, Sedor & Fernandes, 2012). Thus, at present, the “Pirambóia Formation” contains the only valid record of *Chelichnus* in Brazil.

***Chelichnus bucklandi*Jardine, 1850**

**Referred material.** SLIA-2 (UFRGS-PV-0601-P), a set of 14 consecutive footprints; SLIA-5 (UFRGS-PV-0602-P), a small slab of tracks in cross-section. The silicon mold UFRGS-PV-0392-P includes the trackway SLIA-2.

**Horizon and locality.** Ibicuí d’Armada locality (21J 0687503/6600663), Santana do Livramento municipality, southwestern region of the Rio Grande do Sul State, southern Brazil; “Pirambóia Formation,” Lopingian–Induan of the Paraná Basin.

**Revised diagnosis.** *Chelichnus* in which *pes* length ranges from 10 to 25 mm; *pes* digit base II–IV separation does not exceed 15 mm; mostly digitigrade but also occasionally plantigrade; trackway pattern most strongly influenced by substrate and slope conditions and may become very irregular in preservation; trackway often found preserved as undertracks (after McKeever & Haubold, 1996).

**Figure 6:**
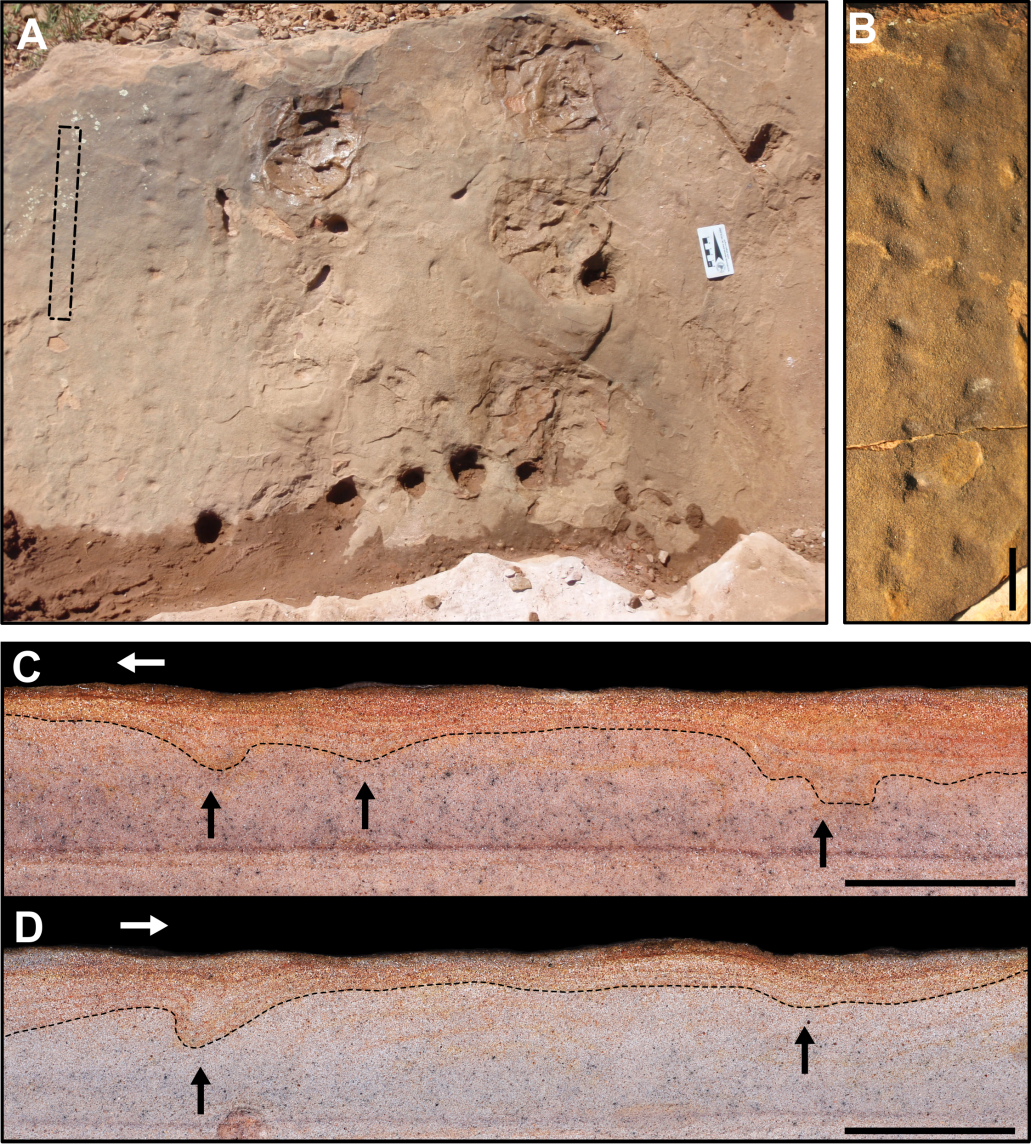
*C. bucklandi* (SLIA-2 and SLIA-5) and indeterminate tracks (SLIA-3) from the “Pirambóia Formation,” Brazil. (A) Plain view of the SLIA site showing the *in situ* position of SLIA-2 (left) and SLIA-3 (right). The intermittent rectangle in the left side of the figure indicates the approximate region in which the slab UFRGS-PV-0602-P (SLIA-5) was collected. (B) Enlarged view of the slab UFRGS-PV-601-P, containing the trackway SLIA-2 after collection. (C) Right side of the slab UFRGS-PV-0602-P (SLIA-5). (D) Left side of the same slab. Black arrows point to the deformation on the sediment caused by the footsteps. White arrows indicate the direction of travel of the trackmaker. Scales: 5 cm (A–B) and 3 cm (C–D).

**Description.** The specimen SLIA-2 (UFRGS-PV-0601-P) is a set of 14 shallow tracks, preserved in concave epirelief (Figs. 6A–6B). Trackway with a wide gauge and marked homopody (i.e, the *manus* and *pes* are dimensionally and morphologically the same; Leonardi, 1987). The mean values of the oblique pace length, pace angulation and stride length are about 66.3 mm, 83.5° and 84.5 mm, respectively (Table S3 in the Supplemental Information File). The mean internal and external trackway widths are about 12.9 and 74.8 mm.

The autopodia are wider than long (width/length ratio about 1.64) and have an elliptical shape (Table S4 in the Supplemental Information File). The digit imprints are not preserved, but paraxonic or mesaxonic conditions are inferred by the oval shape of the tracks. Some autopodia are oriented inwards (about 21°). The digits cannot be recognized in any track, and tail- or body-drag traces are not present. Displacement rims of sediment and “sand crescents” are not present.

The gleno-acetabular distance inferred for the SLIA-2 trackmaker is about 121.4 or 78.9 mm, considering alternative walk or amble gaits, respectively.

SLIA-5 (UFRGS-PV-0602-P) preserves some indeterminate autopodia imprints in cross-section, easily recognized by the folded laminae of the substrate (Figs. 6C–6D). They are concave up, about 15 mm long, and the deformed layers are 6 mm deep. On one of the sides of the slab, it is possible to see two potential consecutive tracks that are 108.9 mm apart. Despite the lack of morphological details, the measurements of these tracks are in accordance with those expected for *C. bucklandi.* Therefore, we attribute the tracks preserved in cross-section on the slab UFRGS-PV-0602-P to this ichnospecies. The level in which these tracks were produced is 8.2 mm below the SLIA-1 and SLIA-2 level, so we consider them contemporaneous.

**Figure 7:**
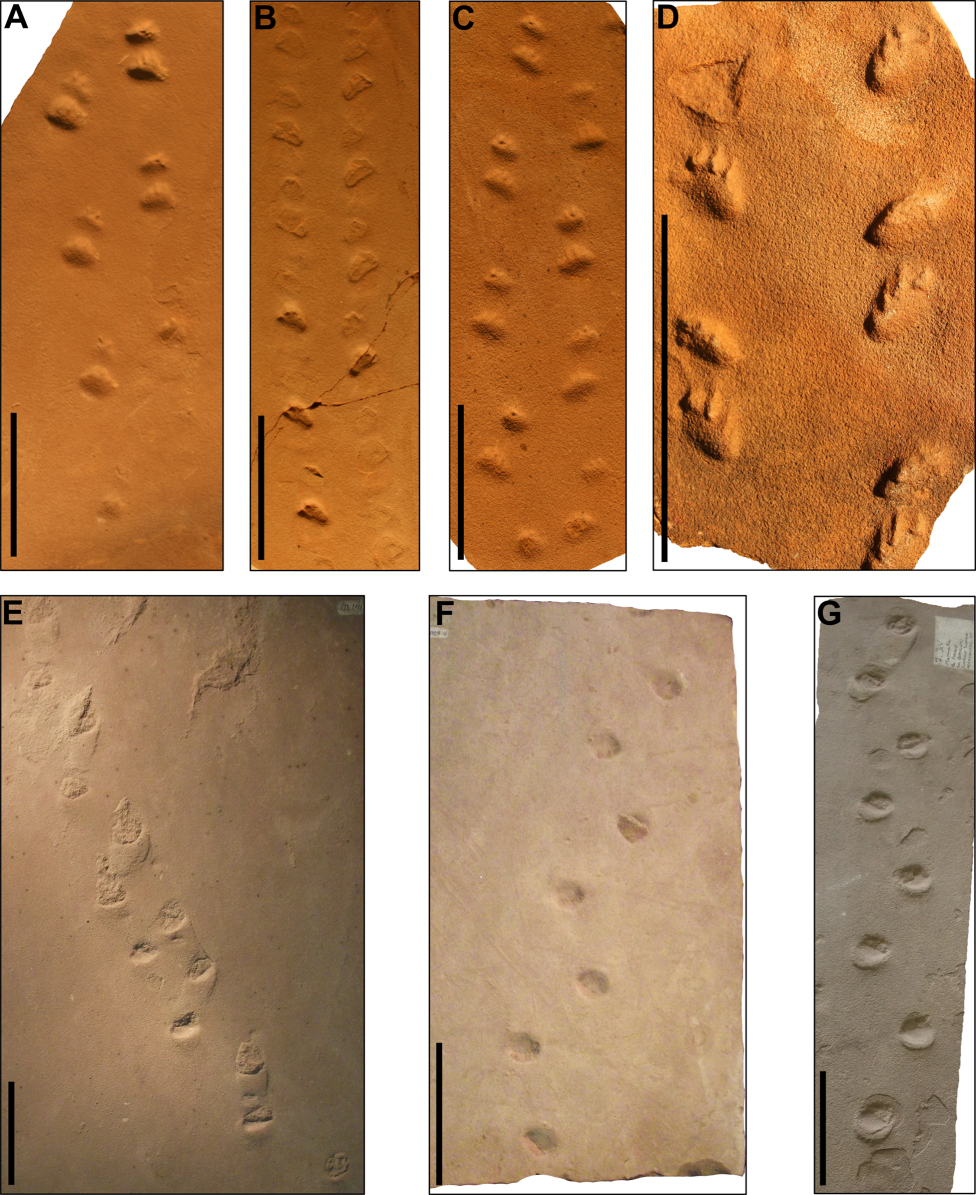
*Chelichnus bucklandi* (A–D) and *Brasilichnium elusivum* (E–G) tracks. (A) Specimen MNA-V3331. (B) Specimen MNA-V3338. (C) Specimen MNA-V3349. (D) Specimen MNA-V3456. (E) MN-3902-V (Holotype). (F) Specimen MN-3903-V (Paratype). (G) Specimen UFRJ-007-IcV. (A–C) from the Coconino Sandstone (Cisuralian of the United States). (D) from the DeChelly Sandstone (Cisuralian of the United States). (E–G) from the Botucatu Formation (Lower Cretaceous of Brazil). Scales: 10 cm.

**Remarks.** SLIA-2 has several features that often occur in chelichnopodid trackways with an uphill travel direction, such as: alternate gait, notable homopody, wider than long autopodia with inward rotation and absence of distinct digits and sole/palm pads. These characters are present in several *C. bucklandi* tracks from the Coconino Sandstone (such as MNA-V3331, MNA-V3338 and MNA-V3349), DeChelly Sandstone (such as MNA-V3456) and *Brasilichnium elusivum* tracks from the Lower Cretaceous Botucatu Formation of Brazil (such as the type-materials MN-3902-V and MN-3903-V) (Fig. 7). However, *B. elusivum* has marked heteropody (i.e., *manus* and *pes* are dimensionally and/or morphologically different; Leonardi, 1987) and *manus* imprints are not that often preserved or shallowly imprinted (Fig. 7), so the SLIA-2 tracks are closely more similar to *C. bucklandi.*

As discussed by Loope (1986), tetrapod tracks in cross-section are easily misinterpreted as non-biogenic deformation structures such as lateral compression or convolute bedding. However, convolute bedding is an indication of rapid deposition, so it is not congruent with the eolian strata in which the tracks were found (Loope, 1986; Collinson & Thompson, 1982). Lateral compression structures in sand tend to have a large size and are not common in strata deposited by grain saltation (Loope, 1986; McKee, Douglass & Rittenhouse, 1971). According to Mancuso et al. (2016), the biogenic origin of the structures in cross section from the Areniscas Altígradas Member of the Yacimiento Los Reyunos Formation in Argentina were justified by their frequency in size and regular shape, features also observed in the Brazilian materials. Additionally, the size of the cross-section structures described here (SLIA-5; UFRGS-PV-0602-P) is in accordance with the *C. bucklandi* tracks from the same strata (i.e., SLIA-2). Also, given that the tracks recorded in the bedding plane do not show any sort of compression or deformation, this is strongly indicative that the eolian strata of the Ibicuí d’Armada locality were only disturbed by biogenic activity.

**Indeterminate tracks**

**Referred material.** SLIA-3, a set of six consecutive footprints; SLIA-4, a set of 22 tracks. Both are recorded in the silicon mold UFRGS-PV-0392-P.

**Horizon and locality.** Ibicuí d’Armada locality (21J 0687503/6600663), Santana do Livramento municipality, southwest region of Rio Grande do Sul State, southern Brazil; “Pirambóia Formation,” Lopingian–Induan of the Paraná Basin.

**Description.** The trackway SLIA-3 is a set of six tracks that occurs in the same bedding plane as SLIA-2 (*C. bucklandi;* Figs. 3B and 6A). All the tracks are poorly preserved so they could not be assigned to an ichnotaxon. The mean length and width of the tracks are 165 and 157.5 mm, respectively, representing the largest tracks recorded in the Ibicuí d’Armada outcrop. The width/length ratio is 0.95, and the distance of the tracks from the trackway midline is 100.6 mm (Table S5 in the Supplemental Information File). Oblique pace length and stride length reach 416 and 315 mm, respectively (Table S6 in the Supplemental Information File).

**Figure 8:**
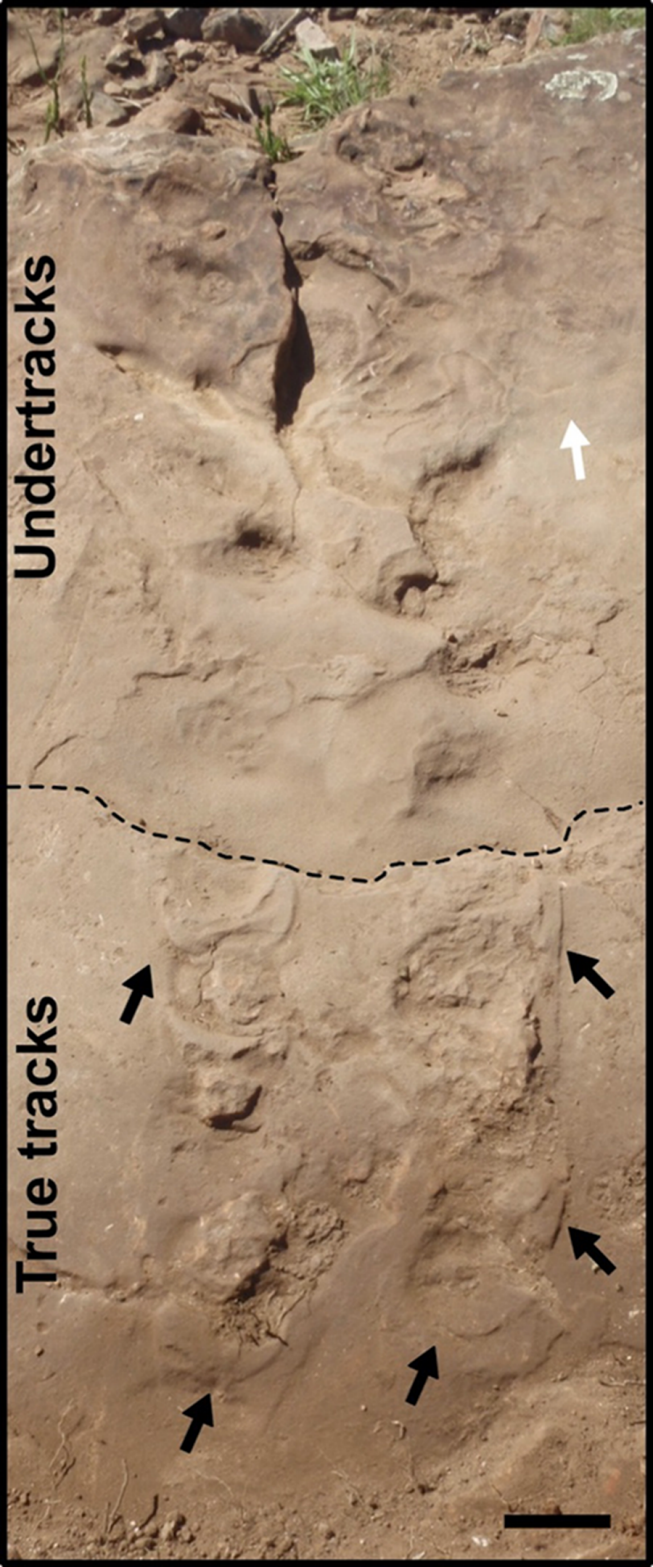
Indeterminate tracks (SLIA-4) from the “Pirambóia Formation,” Brazil. The dashed line indicates a fracture in the substrate, causing differences in the preservation of the tracks. Black arrows indicate the displacement rims on the posterior margins of the true tracks and the white arrow indicates the direction of the trackmaker’s travel. Scale: 15 cm.

The trackway SLIA-4 is a set of 22 tracks (Figs. 3B and 8) whose limits and dimensions are difficult to identify. The *manus* and *pedes* are not well enough preserved for us to be able to characterize them. Although SLIA-4 shares with SLIA-1 the same bedding plane, size and travel direction, it is poorly preserved, preventing an accurate ichnotaxonomic assignment. Additionally, there is a fracture that exposes the sandstone layer below that in which the original tracks were produced, so that almost two-thirds of the SLIA-4 tracks are preserved as undertracks (Fig. 8). In spite of being at the same level as SLIA-1, the original SLIA-4 (the remaining one-third) tracks present displacement rims positioned in the posterior margin of the autopodium impressions.

## Discussion

### Ichnotaxonomic comparison

The specimens described here as *Dicynodontipus* isp. and *C. bucklandi* share several morphological features with other ichnotaxa, mainly *C. duncani* Jardine, 1850, *Brasilichnium* Leonardi, 1981, *Navahopus* Baird, 1980 and some material attributed to *Dicynodontipus* Rühle von Lilienstern, 1944.

**Figure 9:**
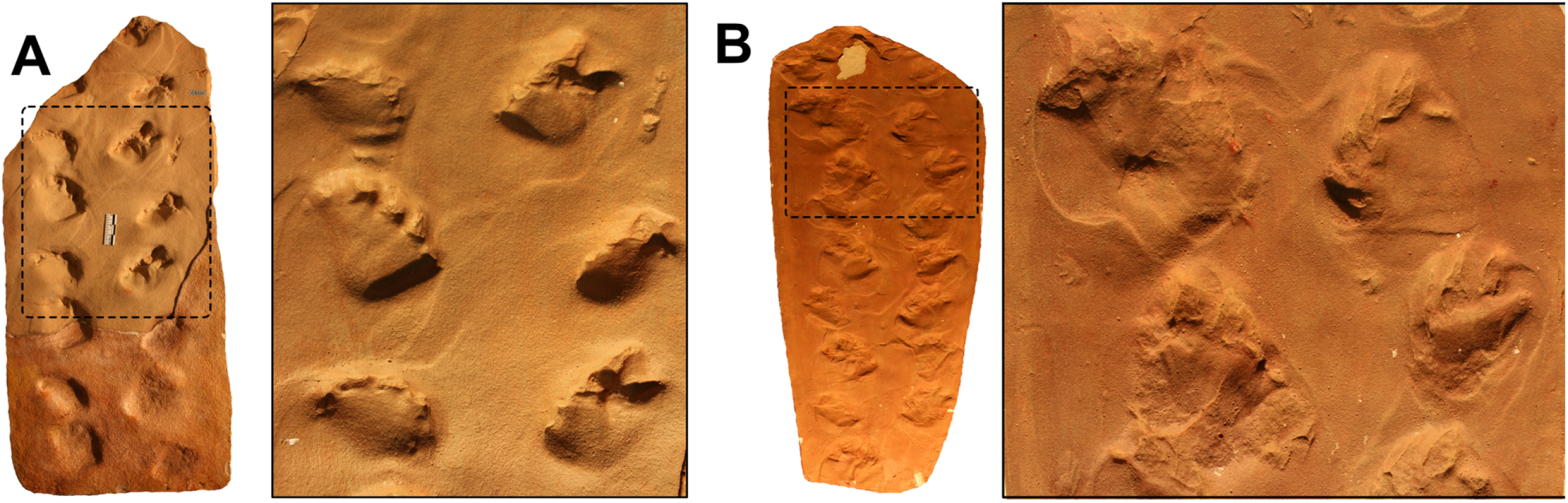
*Chelichnus duncani* trackways from the Coconino Sandstone (A) and the DeChelly Sandstone (B) of the United States. (A) Holotype of “*Baropezia eakini*” (USNM-11137). (B) “*Agostopus matheri*” (MNA-V1556).

Triangular-shaped tracks (similar to SLIA-1) occur in materials attributed to *C. duncani*. For example, the material proposed by Gilmore (1926) to be the holotype of “*Baropezia eakini*” (USNM-11137; now considered to be a junior synonym of *C. duncani*; Fig. 9A; McKeever & Haubold, 1996) has deep tracks, with subtriangular *pedes* and a suboval *manus*, which are evident in both part and counterpart. The specimen USNM-11138 (formerly, the paratype of “*B. eakini*”) also presents the same morphology, but due to its suboptimal preservation, the complete shapes of the tracks are not so evident. However, the SLIA-1 tracks are different from “*B. eakini*” mainly in the forward orientation of the *pes* tracks and the manual digitigrady. In addition, the specimen USNM-11137 has rounded digits (Fig. 9A), which is very different from the typical *Chelichnus* tracks, but more like the drumstick-shaped digits of *Ichniotherium* (Voigt, Berman & Henrici, 2007). In USNM-11138, the right digit traces are longer and seems to form drag marks (Fig. 9B). Notwithstanding, the similarity between the triangular shape of “*B. eakini*” and SLIA-1 tracks, the digit configuration of the former prevents an assignment of the Brazilian tracks to *C. duncani*.

The SLIA-1 tracks are also close in morphology to *C. duncani* (=“*Agostopus matheri*”) from the DeChelly Sandstone of Arizona, mainly with those where the travel orientation is straight uphill (such as MNA-V1556; Fig. 9B), which have anteriorly or slightly inward oriented autopodia and short strides. However, some specimens (such as MNA-V3442) have particular features that result from the change in the gait adopted during downhill locomotion on dunes, such as strong inward rotation of both *manus* and *pedes*, incomplete palm and sole imprints, long strides and digit drag traces (Morales & Haubold, 1995). Even though the SLIA-1 trackway is clearly oriented uphill, its manual tracks are also rotated inwards, whereas the feet point anteriorly.

*Brasilichnium* also comprises quadrupedal, heteropod, synapsid-related tracks with a rounded to transversely oval shape, being grouped with *Chelichnus* under the ichnofamily Chelichnopodidae (Fig. 7; Leonardi, 1981; Fernandes & Carvalho, 2008; Lockley, 2011). However, they differ mainly by the digit count (*Chelichnus* is pentadactyl, though *Brasilichnium* is tetradactyl) and by the marked heteropody presented by *Brasilichnium* (Leonardi, 1981; Fernandes & Carvalho, 2008; Lockley, 2011). The ichnogenus *Brasilichnium* was initially erected to describe only one of the mammaloid track morphotypes from the Lower Cretaceous Botucatu Formation of Brazil (Fig. 7; Leonardi, 1981), but it was also recognized in several eolian and non-eolian units throughout the Mesozoic of Brazil, Namibia and the United States (Hunt & Lucas, 2006; Fernandes & Carvalho, 2008; Lucas et al., 2010; Lockley, 2011; Porchetti & Wagensommer, 2015). *Brasilichnium* is also known by its wider temporal range (Late Triassic–Late Cretaceous), contrasting with *Chelichnus*, which is confined to the Permian (McKeever & Haubold, 1996). However, both ichnogenera do not occur in the same strata, and there is a gap without chelichnopodid tracks between the latest Permian and the Late Triassic. Therefore, both morphology and temporal range are in favor of an interpretation of the SLIA-2 tracks as belonging to *Chelichnus*.

**Figure 10:**
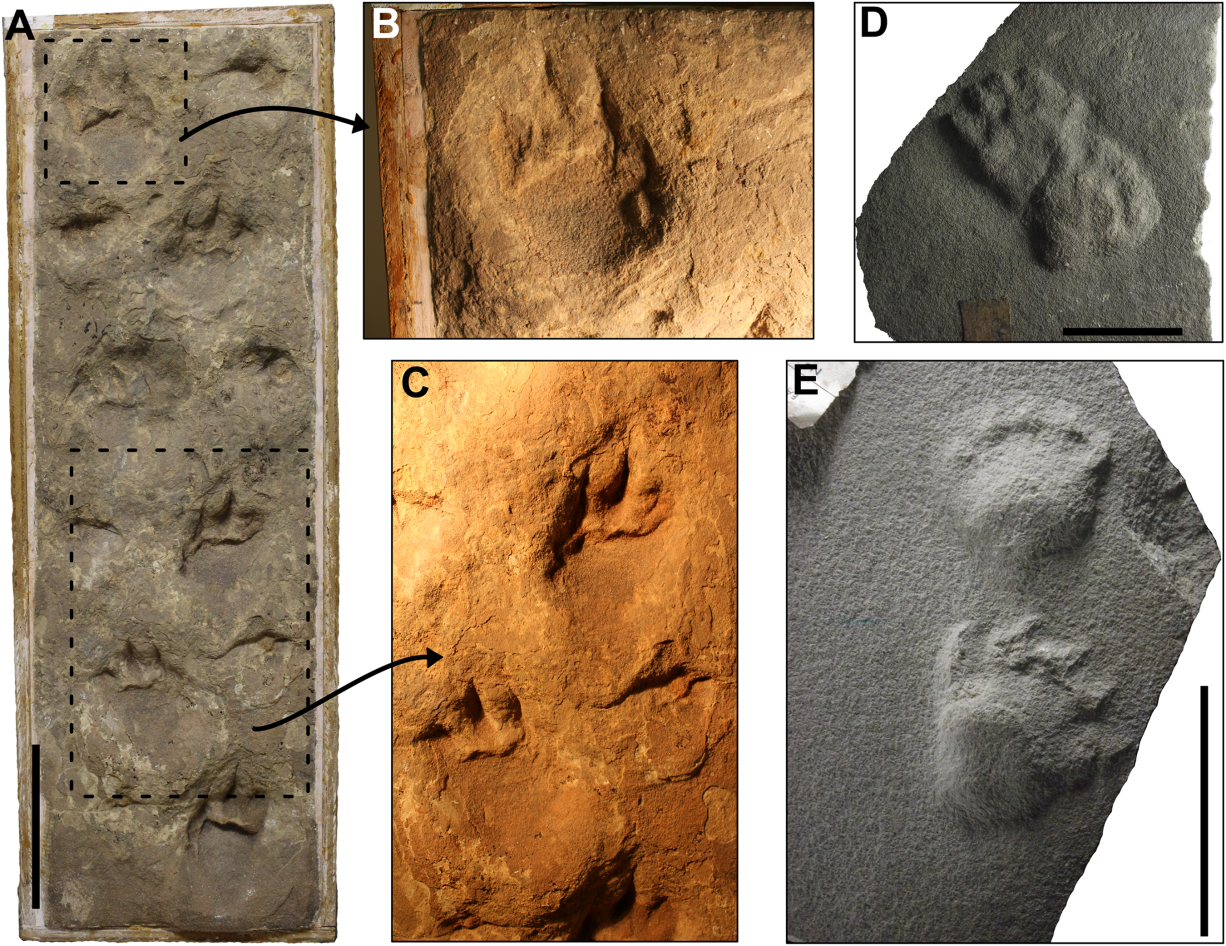
*Navahopus falcipollex* (A–C) and *Dicynodontipus* ispp. (D–E) tracks. (A) Specimen MNA-V3430 (Holotype) from the Navajo Sandstone (Lower Jurassic of the Unites States). (B–C) Details of the same specimen. Note the marked heteropody and the tetradactyly in *Navahopus*, but absent in the “Pirambóia” tracks. (D) Specimen MLP-66-XI-15-3 (“*Gallegosichnus garridoi*”). (E) Specimen MLP-60-XI-31-4 (“*Calibarichnus ayesterani*”). (D–E) from the Vera Formation (Upper Triassic of Argentina). Scales: 15 cm (A) and 5 cm (D–E).

Another ichnotaxon that is similar to the tracks described here is the poorly known *Navahopus falcipollex*, from the Lower Jurassic Navajo Sandstone of the western USA (Figs. 10A–10C; Baird, 1980; Hunt & Lucas, 2006). Although the validity of this ichnotaxon was disputed because it is known only by its suboptimally preserved type material (MNA V3430; Lockley & Hunt, 1995; Lockley et al., 1995; Lockley & Tedrow, 2009), it is currently considered valid (Hunt & Lucas, 2006; Lockley, 2011). *N. falcipollex* was described by Baird (1980) as tracks of a quadrupedal, heteropodous and tetradactyl animal with falciform pollexes that are directed inwards. But, according to Hunt & Lucas (2006), this latter character is more likely an extramorphological feature, an opinion that is closely followed here. The reinterpretation of the claw traces of *Navahopus* approximates this ichnogenus morphologically to the Chelichnopodidae. Actually, several authors noted the similarity between *Navahopus* and *Brasilichnium* (Lockley & Hunt, 1995; Hunt & Lucas, 2006; Reynolds, 2006; Lockley & Tedrow, 2009; Lockley, 2011), including Milàn, Loope & Bromley (2008), who reinterpreted a trackway previously attributed to *Brasilichnium* by Loope & Rowe (2003) as a different ichnospecies of *Navahopus* (*N. coyoteensis*). However, *N. falcipollex* has *pes* imprints that are longer than wide, different from the usually wider than long *pes* tracks of *Chelichnus* and *Brasilichnium* (Hunt & Lucas, 2006). Notwithstanding, this feature is also present on the SLIA-1 trackway, which differs from *N. falcipollex* mainly in the pedal digit count and the divarication of the *manus* (inward directed in the Brazilian tracks). Also, the “Pirambóia” tracks have more defined triangular-shaped feet, contrasting with the triangular to rounded *pedes* of *N. falcipollex*, which have been more influenced by extramorphological variation.

In addition to the *Brasilichnium* classic gait, represented by its type ichnospecies *B. elusivum* Leonardi (1981), two other ichnospecies were recently erected: *B. saltatorium* Buck et al. (2017a), which is represented by the hopping variation in the gait of the same producer of *B. elusivum*; and *B. anaiti* Porchetti, Bertini & Langer (2017), a supposed large form of *Brasilichnium*. Porchetti, Bertini & Langer (2017) noted that *B. anaiti* is extremely similar to *Navahopus* but they were not able to stress the differences between both ichnotaxa.

Simultaneously, Buck et al. (2017b) described the same material as belonging to a new monospecific ichnogenus, *Aracoaraichnium leonardii,* but they also ignored *Navahopus.* Although it is not within the scope of this contribution to revise these newly proposed ichnotaxa (Buck et al., 2017b; Porchetti, Bertini & Langer, 2017), we are confident that *B. anaiti* and *A. leonardii* are junior subjective synonyms of *Navahopus*, especially because the morphology of both is very much influenced by the extramorphological features related to walking on eolian sands (e.g., the manual digit count and variation of the *manus* shape) and it is not possible to differentiate the anatomical differences between their trackmakers. As explained above, the morphology of the SLIA-1 tracks indicates that it is closely related to *Navahopus, B. anaiti* and *A. leonardii*. However, the pentadactyly and the inward rotation of the *manus* imprints (not seen in *N. falcipollex, B. anaiti* and *A. leonardii*) are sufficient to differentiate them and place SLIA-1 within the ichnogenus *Dicynodontipus*.

Besides the typical ichnotaxa of the *Chelichnus* Ichnofacies (i.e., *Chelichnus, Brasilichnium* and *Navahopus*), the most similar tracks are some of those described by Casamiquela (1964, 1975) from the Upper Triassic volcaniclastic Vera Formation of the Los Menucos Depocentre (Río Negro Province) of Argentina (Figs. 10D–10E). Originally, Casamiquela (1964, 1975) recognized four theromorphoid ichnotaxa (“*Calibarichnus ayesterani*,” “*Gallegosichnus garridoi*,” “*Palaciosichnus zetti*” and “*Stipanicichnus bonnetti*”) that are currently interpreted as belonging to four different ichnospecies of *Dicynodontipus* (Melchor & de Valais, 2006). Two of these ichnotaxa (“*C. ayesterani*” and “*G. garridoi*”) are represented by triangular, pentadactyl footprints with short and broad digits, which are clearly similar to the SLIA-1 tracks of Brazil (Figs. 10D–10E).

The type materials of “*Calibarichnus*” and “*Gallegosichnus*” were interpreted originally as being produced by the right autopodia of the trackmaker (Casamiquela, 1964; Melchor & de Valais, 2006), which implies an outward rotation of the *manus*. However, Leonardi & Oliveira (1990) and Domnanovich et al. (2008) described new, more complete material from the Cerro de las Lajas locality, with a clear inward position of the *manus* with respect to the track midline. According to these authors, the foot imprints are oriented forward (Domnanovich et al., 2008). These features are also seen in the tracks attributed to “*Gallegosichnus*” (Casamiquela, 1964; Leonardi & Oliveira, 1990; Domnanovich & Marsicano, 2006; Domnanovich et al., 2008), although the opposite pattern was proposed by Melchor & de Valais (2006). In a general overview, both “*Calibarichnus*” and “*Gallegosichnus*” share several characters with the SLIA-1 tracks: these quadrupedal trackways are composed of an inward oriented *manus* placed anterior to the pentadactyl, nearly mesaxonic, plantigrade, forward oriented and triangular-shaped *pes* imprint (Figs. 10D–10E). Because the Los Menucos tracks preserve several fine details (such as digits and sole/palm pads), we understand that they reliably represent the anatomy of the trackmakers, and their similarity to the SLIA-1 tracks should correspond to the anatomical similarity of the producers.

*Dicynodontipus hildbughausensis* and *D. geinitzi*, from the Early Triassic of Germany, also shares several features with SLIA-1 (Hornstein, 1876; Rühle von Lilienstern, 1944). *D. geinitzi* is an ichnotaxon of a quadrupedal, homopod animal, with the phalangeal formula 2-3-3-3-3 (Rühle von Lilienstern, 1944; Haubold, 1965). The *manus* is rotated inwards (30°–40°) and the *pedes* are positioned parallel to the trackway midline (Rühle von Lilienstern, 1944), as occur in the Ibicuí d’Armada tracks. Nevertheless, in contrast to the German material, the “Pirambóia” tracks have only one metacarpal pad preserved in each *pes*, a condition equivalent to the *Dicynodontipus* ispp. (i.e., “*Calibarichnus*” and “*Gallegosichnus*”) from Argentina.

Citton et al. (2017) have noted the similarity between *D. geinitzi* and *Contiichnus tazelwurmi* (Lopingian of Italy), this latter originally described as *“Chelichnus” tazelwürmi*. As stated by these authors, *C. tazelwurmi* differs from *Chelichnus* mainly because of its strong heteropody, manual entaxony, the triangular shape of the *pes* and the continuity between the sole/palm print and the digit prints (Citton et al., 2017; Bernardi et al., 2017). On the other hand, Marchetti, Voigt & Klein (2017) consider that this material belongs to the ichnogenus *Procolophonichnium* (Table 2).

Among other *Dicynodontipus* ichnospecies, *D. icelsi* from the late Permian Asante Sana paleosurface in the *Cistecephalus* Assemblage Zone of the Karoo Basin (South Africa) is the ichnotaxon that shares more features with SLIA-1 (De Klerk, 2002). De Klerk (2002) described *D. icelsi* based on seven trackways (H, J, K, N, P, Q and Z) produced by a quadrupedal and heteropodous, medium-sized animal. The *manus* and *pedes* of *D. icelsi* are pentadactyl, plantigrade and wider than long (De Klerk, 2002), similar to SLIA-1 from Brazil. However, *D. icelsi* has long digit imprints (De Klerk, 2002), which do not occur in the Brazilian tracks. The trackway also has an alternate gait, and both autopodia vary in divarication, being inward oriented (as in the trackway Z) or with the main axis parallel to the trackway midline (as in the trackway Q). Additionally, *D. icelsi* has well marked and rounded pedal pads, different from the SLIA-1 pads, which give a triangular shape to the feet. Although SLIA-1 has the same alternating gait, its *manus* imprints are always oriented inward, at about 30°, whereas its *pedes* imprints point forward. However, as seen in several *Chelichnus* materials (e.g., the specimens RAM 123 and RAM 131) from the Coconino Sandstone, autopodium divarication can change in response to the variation of dune slopes and the direction of travel relative to the dune crest. As discussed below, the ichnotaxonomy of these tracks formerly assigned to *Dicynodontipus* is not universally accepted and Marchetti, Voigt & Klein (2017) assigned them to *Dolomitipes* isp. (Table 2).

Another large *Dicynodontipus* track is *D. bellambiensis* from the Lower Triassic Coal Cliff Sandstone of Australia (Retallack, 1996). However, despite its size and Early Triassic age, it is very different from SLIA-1, mainly in having long (19–54 mm) and much divaricated digits (about 65°–133°) and a forward oriented *manus* (Retallack, 1996).

The trackway SLIA-1 also shares several features with *Pachypes*, a pareiasaur-related ichnogenus from the Lopingian Val Gardena Sandstone and Bellerophon Formation of Italy and the Ikakern Formation of Morocco (Leonardi et al., 1975; Valentini, Conti & Nicosia, 2008; Valentini, Nicosia & Conti, 2009; Voigt et al., 2010). Both have a strongly inward turned *manus* and forward directed *pedes*, with well-developed sole impressions (Leonardi et al., 1975; Valentini, Nicosia & Conti, 2009). However, the *pedes* of *Pachypes* are markedly ectaxonic (i.e., the most important digit is the digit IV; Leonardi, 1987), with a small digit V that is consistent with pareiasaurian foot morphology (Valentini, Conti & Nicosia, 2008), whereas the mesaxonic *pedes* of SLIA-1 fit better with therapsid morphology (see below). Additionally, it is important to mention the similarity between the alternate gait of SLIA-1 and TW-1, a trackway first described as “*Sukhonopus*” by Gubin & Bulanov (in Gubin et al., 2003) and later synonymized with *Pachypes* (Valentini, Conti & Nicosia, 2008). According to Gubin & Bulanov (in Gubin et al., 2003), *manus* and *pedes* of “*Sukhonopus*” are arranged in the alternately opposite pattern of Haubold (1971b), a pattern also observed in SLIA-1. However, this gait pattern is not ichnotaxonomically diagnostic, because several quadrupedal animal taxa can produce alternating trackways when they walk with normal paces (*sensu* Leonardi, 1987), including the *Chelichnus* trackmakers (see examples in Gilmore, 1926).

Another medium- to large-sized Permian ichnogenus is *Brontopus* from the upper Permian of the Lodève Basin of France (Heyler & Lessertisseur, 1963; Gand et al., 2000). Although Gand et al. (2000) have compared this French ichnotaxon with *Chelichnus* from the Elgin area of Scotland and considered them very similar in size and morphology, they maintained both ichnogenera as valid taxa. *Brontopus* digits decrease in size from II to V, and the digit I is the smallest (Gand et al., 2000). This pattern led Gand et al. (2000) to attribute this ichnogenus to dinocephalians, even though late Permian dicynodonts, therocephalians and eucynodonts also have nearly symmetrical, mesaxonic autopodia with digit I smaller than the more external digits (Hopson, 1995). The SLIA-1 *pes* also has this pattern, but the *manus* differs from *Brontopus* in proportions (the *manus* of *Brontopus* has a nearly equal length and width).

Some Mesozoic synapsid ichnogenera compose the ichnofamily Ameghinichnidae Casamiquela, 1964, represented by *Ameghinichnus* from the Middle Jurassic La Matilde Formation of Argentina and *Catocapes* from the Lower Cretaceous Continental Intercalaire Group of Angola (Casamiquela, 1964; De Valais, 2009; Mateus et al., 2017). Although these ichnogenera also are represented by nearly homopodous, plantigrade, mesaxonic and wider than long and pentadactyl tracks, there are several differences between them and the material described here. Because these ameghinichnid tracks were produced on fine sediments, they have preserved fine details that clearly are related to the producer’s anatomy. For example, the manual and pedal digits of *Ameghinichnus* and *Catocapes* are widely divaricated, reaching 151° in *A. patagonicus* (Casamiquela, 1964; De Valais, 2009; Mateus et al., 2017), whereas the digits in *Dicynodontipus* and *Chelichnus* (including SLIA-1 and SLIA-2, respectively) are mostly forward directed and less divaricated. Other important differences are the sinuous and continuous tail traces associated with *Ameghinichnus* autopodia and its outward rotated feet, which can be observed in the alternating and opposite arrangements of the *manus*-*pes* sets (Casamiquela, 1964; De Valais, 2009).

Hunt et al. (1993) described some trackways from the Middle Triassic Holbrook Member of the Moenkopi Formation (Arizona, USA), naming them *Therapsipus*. This ichnogenus is represented by large quadrupedal animal tracks in an alternating pattern, but the outward direction of both the *manus* and the *pes* imprints is very different from the morphology observed in SLIA-1 (Hunt et al., 1993).

Based on the information provided above, the SLIA-1 and SLIA-2 tracks from the “Pirambóia Formation” have a high morphological affinity with the ichnogenera *Dicynodontipus* (mainly the materials from Germany and Argentina) and *Chelichnus* (materials from Scotland, Germany, the USA and Argentina). However, several ichnotaxa have been synonymized with *Dicynodontipus* and *Chelichnus* (Tables 1 and 2; e.g., McKeever & Haubold, 1996) and now they are known by many morphological and extramorphological variations that make difficult the understanding of the real ichnotaxonomic meaning of their diagnoses. A comprehensive ichnotaxonomic revision of the ichnogenera *Dicynodontipus* and *Chelichnus* is imperative to allow the recognition of these ichnotaxa in other deposits and avoid mistakes in ichnostratigraphic studies and correlations between the track and their trackmakers. However, even knowing this problem, we opted to attribute the “Pirambóia Formation” tracks to *Dicynodontipus* isp. (SLIA-1) and *C. bucklandi* (SLIA-2 and SLIA-5), since they are very close in morphology. Future advances in the understanding of extramorphological variation among the tracks produced in eolian deposits and in the ichnotaxonomy of these ichnogenera should shed additional light on this issue.

### The trackmackers’ identities

The morphology of SLIA-1 indicates that its producer was a quadrupedal, pentadactyl and middle-sized animal, with nearly symmetrical feet and short, subequal digits. This morphology is very different from temnospondyl amphibian tracks (e.g., *Batrachichnus*, *Palaeosauropus* and *Limnopus*), because they have ectaxonic *pedes* and a tetradactyl *manus* (Marsh, 1894; Baird, 1952; Turek, 1989; Haubold et al., 1995; Melchor & Sarjeant, 2004; Marsicano, Wilson & Smith, 2014).

Another middle- to large-sized group of animals that lived in Guadalupian–Lopingian environments is Pareiasauria (e.g., Cisneros, Abdala & Malabarba, 2005 and references therein). Short, broad digits with the pedal phalangeal formula 2-3-3-4-3 and the fusion between astragalus and calcaneum are apomorphies that define the taxon Pareiasauroidea (Pareiasauria + *Sclerosaurus*) (Romer, 1976; Jalil & Janvier, 2005; Valentini, Conti & Nicosia, 2008; Valentini, Nicosia & Conti, 2009). Additionally, pareiasaurs have small pedal fifth digits, which are shorter than or as large as the hallux (Jalil & Janvier, 2005). Leonardi et al. (1975) were the first to relate the pareiasaur autopodium anatomy to footprints from the Lopingian Val Gardena Sandstone (Italy), naming them *Pachypes dolomiticus*. Later, other *Pachypes* materials were described from Italy, Russia and Morocco, and the affinity between this ichnotaxon and pareiasaurs was strengthened (Gubin et al., 2003; Valentini, Conti & Nicosia, 2008; Valentini, Nicosia & Conti, 2009; Voigt et al., 2010).

As mentioned above, we noted some similarities between SLIA-1 and the trackway TW-1 from the late Permian (*Proelginia permiana* Zone, Severodvinian Horizon) of Russia. This trackway was first described as “*Sukhonopus*” (Gubin et al., 2003), but Valentini, Conti & Nicosia (2008) considered it to belong to *Pachypes*. The Russian trackway TW-1 is composed of triangular-shaped *pedes* with short digits and an elliptical *manus*, grouped in a “reciprocal opposed” condition (*sensu* Haubold, 1971b), similar to SLIA-1 (Gubin et al., 2003). However, Voigt et al. (2010) argued that the Russian material is not sufficiently well-preserved to confirm its attribution to *Pachypes* and, as a consequence, to pareiasaurs. Therefore, based on the morphological differences between *Pachypes* and the trackways described here (i.e., the typical ectaxonic configuration of *Pachypes* with diminutive pedal digit V) and the lack of confidence in the pareiasaurian affinity of the Russian tracks, we conclude that the Ibicuí d’Armada trackways were not produced by Pareiasauria.

Late Permian–Early Triassic archosauromorph tracks (e.g., *Protochirotherium*) show a unique morphology, with a pedal digit V strongly reduced and posterolaterally positioned and a digit IV shorter than or as long as digit III (e.g., Conti et al., 1977; Mietto & Muscio, 1987; Klein et al., 2013; Bernardi et al., 2015). These characters are considered archosauromorph apomorphies and can be traced in several Permo–Triassic species, such as the archosauriform *Euparkeria* and erythrosuchians (Klein et al., 2013; Bernardi et al., 2015). The SLIA-1 *pedes* are mesaxonic and show forward directed digits that are very similar in size, which is contrary to archosauromorph foot morphology.

On the other hand, late Permian–Early Triassic therapsid synapsids have more symmetrical, mesaxonic autopodia (Hopson, 1995; Kümmell & Frey, 2012), which corresponds to the morphology of SLIA-1 and SLIA-2. The reduction in the number of phalanges in the third and fourth manual and pedal digits from the “pelycosaur” condition (manual and pedal phalangeal formulae 2-3-4-5-3 and 2-3-4-5-4, respectively) to the mammalian condition (both manual and pedal phalangeal formulae 2-3-3-3-3) was a transition that occurs convergently among the major groups of therapsids (Hopson, 1995), giving a more symmetrical shape to their autopodia. The therapsid feet are plantigrade, and some advanced taxa (mostly therocephalians and cynodonts) have a posterior border of the calcaneum forming a projection (the *tuber calcis*) for the insertion of the distal tendons of the *musculus gastrocnemius* and other calf muscles (e.g., Jenkins, 1971; Kemp, 1982; Szalay, 1993; Oliveira, Soares & Schultz, 2010 and references therein). However, some taxa seem to not have an ossified *tuber calcis*, such as the basal cynodont *Thrinaxodon* (Jenkins, 1971). On the other hand, the therapsid *manus* is more conservative, in spite the tendency to lose the phalanges (Kemp, 1982). The mammalian condition of pollex divergence, however, is not widespread among non-mammalian therapsids (Romer, 1976).

According to Kümmell & Frey (2012), the morphology of the metapodial articular heads in non-mammaliamorph therapsids indicates that their main body mass was transferred to the substrate through the distal part of the metapodials and the proximal part of the proximal phalanges. Therefore, the deepest region of the therapsid footprints should be the metapodial-phalangeal articulation, as can be observed in the *pes* prints of the SLIA-1 trackway (Figs. 5D–5G).

The primitive phalangeal formula (2-3-4-5-3) was retained by biarmosuchians and several gorgonopsians, although few complete autopodial skeletons of these therapsids are known (Hopson, 1995; Rowe & van den Heever, 1986). Consequently, we not consider these groups as possible trackmackers of the Ibicuí d’Armada tracks.

Besides some contradictions, all dinocephalians seems to have had the mammalian phalangeal formula (Rowe & van den Heever, 1986). According to Hopson (1995) and Kemp (1982), the carnivoran clade Brithopia (represented by the anteosaurid *Titanophoneus* from the Guadalupian of Russia) had four phalanges in the manual digit IV. However, several authors agree that the phalangeal formula 2-3-3-3-3 is widespread within dinocephalians (Orlov, 1958; Chudinov, 1983; Rowe & van den Heever, 1986). This phalangeal formula is the same as expected for the SLIA-1 trackmaker, making dinocephalians potential trackmakers. However, dinocephalians were completely extinct during the middle Lopingian (e.g., Boonstra, 1971; Rubidge & Sidor, 2001; Pearson et al., 2013; Day et al., 2015b and references therein), and, according to Carrano & Wilson (2001), the temporal distribution of biological taxa can be used to refine the trackmaker identification. Therefore, we prefer to attribute the Ibicuí d’Armada tracks to another group of therapsids (see below) whose temporal range best fits with the Lopingian–Induan “Pirambóia Formation” record.

Among the late Permian–Early Triassic therapsids, dicynodonts, therocephalians and cynodonts were the most abundant. However, Lopingian–Induan taxa of Cynodontia, such as *Procynosuchus* (*manus* 2-3-4-4-3), *Galesaurus* (*manus* 2-3-4?-4-3; *pes* 2-3-4?-4-3) and *Thrinaxodon* (*manus* 2-3-4-4-3; *pes* 2-3-4-4-3), have asymmetrical *manus* and *pedes* (Hopson, 1995) that are not compatible with the mesaxony observed in the “Pirambóia” tracks. More symmetrical autopodia (i.e., phalangeal formulae of 2-3-3-3-3 in both anterior and posterior autopodia) appeared in the clade Eucynodontia, but *manus* and *pes* records are unknown or incomplete in eucynodont taxa older than Early Triassic (Jenkins, 1970, 1971; Hopson, 1995). Therocephalians have the 2-3-3-3-3 phalangeal formula, but their autopodia show asymmetrical metacarpal proportions, in which metacarpal II is smaller than metacarpal IV (Hopson, 1995), indicating a slightly ectaxonic condition.

Regarding the dicynodonts, all the species that have preserved autopodia show both *manus* and *pedes* phalangeal formulae of 2-3-3-3-3 and metacarpals II and IV of very similar length (Watson, 1913, 1960; Cluver, 1978; King, 1985, 1990; Rubidge, King & Hancox, 1994; Hopson, 1995), giving a symmetrical, near mesaxonic condition to their hands and feet, similar to the SLIA-1 and SLIA-2 tracks. Although this morphology is also common among non-dicynodont anomodonts (Hopson, 1995; Cisneros et al., 2015), some of them have an elongated metacarpal IV (e.g., *Galechirus*) or discoidal extra-phalanges in the third and fourth digits (e.g., *Suminia*), making their autopodia more asymmetrical (Fröbisch & Reisz, 2009).

Dicynodonts are known by their dual gait, which resulted from adducted (upright) hind limbs and more abducted (sprawling) fore limbs (Kemp, 1982; King, 1985, 1990; Blob, 2001; Vega-Dias & Schultz, 2004; Fröbisch, 2006; Ray, 2006; Morato et al., 2008). According to Fröbisch (2006), the astragalus and calcaneum morphology of the Triassic kannemeyeriid *Tetragonias* limits the flexibility of the ankle joint, making it unable to rotate. This feature was also observed in the Lopingian *Dicynodontoides* (*=Kingoria*) by King (1985), indicating that movements of its feet were limited to flexion and extension. Similarly, the SLIA-1 trackway was produced by an animal that had forward-directed feet and fore limbs able to rotate inward, which fits with the functional anatomy of some Permian and Triassic dicynodonts (King, 1985; Fröbisch, 2006). The opposite condition was described in *Cistecephalus*, which has a fused astragalus and calcaneum (Cluver, 1978).

The triangular shape of SLIA-1 foot impressions seems to be an anatomical feature of the trackmaker, produced by a posterior expansion of the *pes*. Anatomically, this projection should be related to the *tuber calcis*, which is a mammalian-grade evolutionary acquisition (Szalay, 1993). Nevertheless, several species of dicynodonts (such as *Eodicynodon*, *Lystrosaurus*, *Tetragonias*, *Dinodontosaurus* and *Jachaleria*) have a rounded calcaneum and lack evidence of an ossified *tuber calcis* (Watson, 1913; King, 1991; Rubidge, King & Hancox, 1994; Vega-Dias & Schultz, 2004; Fröbisch, 2006; Morato et al., 2008). In such cases, the insertion of the *musculus gastrocnemius* and the other calf muscles is inferred to have been at the plantar face of the calcaneum, following the sauropsid condition (Haughton, 1864–1866; Fröbisch, 2003; Morato, 2006). The only known exception to this morphology is the calcaneum of *Dicynodontoides* (=*Kingoria*). In this case, the posterior process of the *Dicynodontoides* calcaneum was considered homologous to the mammalian *tuber calcis* (King, 1985). However, in spite of the data provided by King (1985), we follow Fröbisch (2003) and Morato (2006), considering that the insertion site of the *musculus gastrocnemius* was in the plantar face of the foot. In this way, even though the presence of an ossified *tuber calcis* is not an anatomical feature that occurs within the clade Dicynodontia, the postero-plantar region of their feet is considered the main area for the insertion of the *musculus gastrocnemius* via the calcaneum tendon (Fröbisch, 2003, 2006; Morato, 2006). The triangular shape of the SLIA-1 pedal tracks could be derived from the presence of soft tissues in the posterior margin of the *pes*, because it is to be expected that the calcaneum tendon was inserted in the postero-plantar face of the feet.

**Figure 11:**
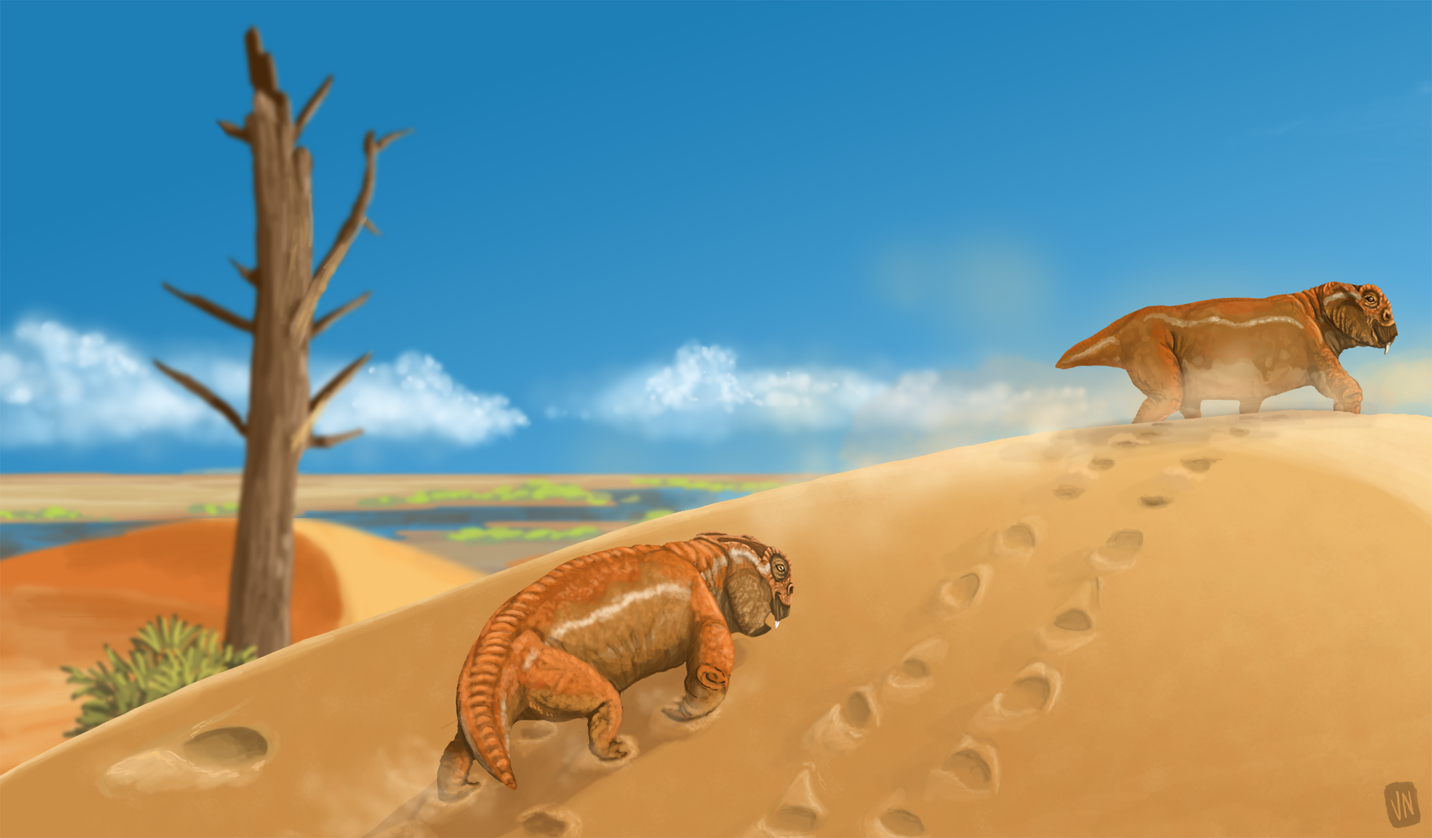
Reconstruction of the Lopingian–Induan “Pirambóia” paleoenvironment at the moment of production of the trackways SLIA-1 and SLIA-4 by two dicynodonts. Image credit: Voltaire Dutra Paes Neto.

Given that SLIA-1 is composed of relatively deep plantigrade footprints (Table S1 in the Supplemental Information File), the gross morphology of its *pes* prints should represent the anatomy of the trackmaker’s feet. This statement is reinforced by the presence of fine details in some pedal tracks, such as the presence of pads. A triangular pedal outline is also observed in some of the *D. icelsi* tracks from the Permian of South Africa and in “*Calibarichnus*” and “*Gallegosichnus*” from the Late Triassic of Argentina. De Klerk (2002) and Kümmell & Frey (2012) related *D. icelsi* to dicynodonts, based on the impressions of the terminal pads of this ichnotaxon, the morphology of the dicynodont autopodia and its coincident occurrence with *Aulacephalodon* and *Dicynodon* in the *Cistecephalus* Assemblage Zone of the Karoo Basin (South Africa). The former ichnogenera “*Calibarichnus*” and “*Gallegosichnus*” from the Vera Formation of Argentina are actually considered synonyms of *Dicynodontipus* by Melchor & de Valais (2006), who attributed these tracks to therapsids. Given the similarity between the Argentinean tracks (Late Triassic) and SLIA-1 (Lopingian–Induan) and their age, here we considered both records as dicynodont related (Fig. 11), also given that this group of therapsids was the only tetrapod lineage with conservative autopodium morphology in this temporal range. The trackway SLIA-4 was produced by a trackmaker of a similar size and, both SLIA-1 and SLIA-4 are parallel tracks produced by animals crossing the dune in the same direction, leading us to hypothesize that they may have been produced by individuals of the same species. However, SLIA-4 lacks morphological details that can corroborate this assumption, and other hypotheses can be raised to explain the co-occurrence of SLIA-1 and SLIA-4 in the same level and the attribution of the latter to a trackmaker.

Regarding SLIA-2 and SLIA-5, both are included in the ichnospecies *C. bucklandi*, which is synapsid related (Lockley et al., 1995 and references therein). Even though these tracks do not preserve the full anatomy of the trackmaker’s autopodia, it is possible to see that their *manus* and *pes* are similar in size and overall shape, different than that expected for “pelycosaur” tracks. These basal synapsids were heteropod and had a sprawling gait, features seen in the “pelycosaur”-related ichnogenus *Dimetropus* (Romer & Price, 1940). Among the synapsids, therapsids are known by their more homopod, symmetrical autopodia and, besides that, there is no record of “pelycosaurs” in the Lopingian (Modesto et al., 2011), favoring the hypothesis of the attribution of SLIA-2 and SLIA-5 to indeterminate therapsids. Other *C. bucklandi* records were attributed to therapsids (Krapovickas et al., 2014), strengthening the coherency of our hypothesis.

### Taphonomy and preservation of the Ibicuí d’Armada trackways

The trackways of the “Pirambóia Formation” occur in two different strata of the same dune with different inclination and preservation. The SLIA-1 and SLIA-4 trackways are associated in the same 20° dipping dune foreset, whereas SLIA-2, SLIA-3 and SLIA-5 occur in another stratum with a 32° dip (Fig. 3). The most well preserved tracks (i.e., those that have more anatomically-related features, such as the presence of digits and pads, in addition to a regular track outline) are those from SLIA-1 that are attributed to *Dicynodontipus* isp. The trackways SLIA-2 and SLIA-5 preserve few morphological characters of the autopodium anatomy of its trackmaker. However, based on their size and the proportion of the autopodia, they can be attributed to *C. bucklandi*. In the case of SLIA-5, the trackway is preserved in cross-section. On the other hand, trackways SLIA-3 and SLIA-4 do not provide sufficient morphological evidence to be attributed to an ichnotaxon nor have their trackmakers been inferred (despite of the discussion above).

Because SLIA-1 and SLIA-4 occur on the same bedding plane that represents a 20° dip dune, we discard the degree of dip of the strata as the main cause of the preservational variation among these trackways. A fracture occurs perpendicular to SLIA-4, exposing part of the undertracks produced during the trackmaker’s progression. On the other hand, some of the true tracks could be recognized by the presence of displacement rims on the posterior margin of some tracks. Based on this and on the absence of these rims in SLIA-1, we assume that the exposed level does not represent the original eolian surface in which the tracks were produced. However, because SLIA-1 is composed of deep tracks, the erosion of the original upper level of the surface did not affect its preservation as in SLIA-4.

In addition, SLIA-1 has an asymmetrical preservation, in which the left set of *manus* and *pedes* preserves more clearly the anatomical details (e.g., digit counts and pedal pads). Intratrackway variations are common in the fossil track record and can be explained by diverse factors, such as variations of substrate consistency and water content (Milàn, 2006; Milàn & Bromley, 2006; Razzolini et al., 2014), abnormal gaits due pathologies and injuries (McCrea et al., 2015; Razzolini et al., 2016), or when the animal is crossing slopes (Razzolini & Klein, 2017). Asymmetrical preservation of trackways (i.e., with one side better preserved than the other) can be observed in several *Chelichnus* tracks from the Coconino Sandstone (e.g., the specimens RAM 247, RAM 382 and RAM 394). We could not relate this variation in preservation to any factor, once the analyzed slabs were *ex situ* (no information about the dune dip was available) and the trackmakers were traveling in different directions (perpendicular, parallel and oblique relative to the dune crest). Nevertheless, these examples from the Coconino Sandstone illustrate that intratrackway variation in preservation is common in the track record of the *Chelichnus* Ichnofacies, including the “Pirambóia Formation” tracks.

Intratrackway variations are also recorded in actual trackways made in eolian environments. For example, tracks produced on the eolian dunes of the Great Sand Dunes National Park (Colorado State, USA) during summer vary enormously in depth and the presence of displacement rims (Fig. S2 in the Supplemental Information File). Even being produced by the same trackmaker (in this case, a human) in strata with the same dip and with no time-averaging, these tracks present different morphologies. This example just shows that the role of the variation of eolian substrate humidity and consistency in trackway preservation is not yet completely understood.

Even though several authors provided important data for vertebrate and invertebrate tracks from controlled experiments (McKee, 1947; Brand, 1979; Davis, Minter & Braddy, 2007; Scott, Renaut & Owen, 2010), the preservational modes of Permian eolian tracks are not yet well understood (Mancuso et al., 2016). Mancuso et al. (2016) recognized five taphonomic modes of preservation (“Modes 1–4” and “Trampling”) for the Yacimiento Los Reyunos Formation (Cisuralian of Argentina) *Chelichnus* tracks. Morphological and extramorphological features (such as shape of the palm outline, connection between the palm and the digits, and presence or absence of digit impressions, claw-drag traces and sedimentary marginal rims) vary among these taphonomic modes, being related to variations in the texture and color of the sediment, lamination type and dip angle of the surface (Mancuso et al., 2016). These authors consider that these variations in the preservation of the tracks are related to substrate consistency and trackmaker speeds. Although the preservation of the Brazilian tracks does not allowed us to replicate the methodology proposed by Mancuso et al. (2016), some information can be discussed.

Substrate consistency depends on its rheology and mechanics and varies with the texture (i.e., size, sorting, sphericity, roundness, etc.) of the sand grains, the mineralogical composition of the clasts and the moisture, but other conditions also affect track preservation in an eolian setting, such as the rapid burial of the perturbed sediment, the dip angle of the substrate, and the moisture content at the exact moment of the production of the tracks (McKee, 1944, 1947; Allen, 1997; Manning, 2004; Milàn, 2006; Milàn & Bromley, 2006; Jackson, Whyte & Romano, 2009; Jackson, Whyte & Romano, 2010; Scott, Renaut & Owen, 2010; Razzolini et al., 2014; Mancuso et al., 2016; Milàn & Falkingham, 2016). Biological (such as the animal’s mass, limb dynamics and the geometry of the autopodia) and ecological (such as the trackmaker’s speed and direction of the travel) variations are also known to affect the preservation of tetrapod tracks (Thulborn, 1990; Falkingham, Margetts & Manning, 2010; Falkingham et al., 2011; Falkingham, 2014; Falkingham, Hage & Bäker, 2014).

However, because the trackways described here occur in two strata that belonged to the same eolian dune, it is here understood that their differences in preservation cannot be explained by variations in the substrate texture, mineralogical composition or dip slope. Neoichnological observations and laboratory-controlled simulations indicate that tracks made on moist substrates preserve more accurately the morphology of the trackmaker’s autopodia rather than completely dry or saturated substrates (Manning, 2004; Milàn, 2006; Jackson, Whyte & Romano, 2009, 2010). Moisture variations could have occurred between the time of deposition of the cross-bedded strata in which the tracks were produced, but given that the Ibicuí d’Armada outcrop is a small exposure and the “Pirambóia” tracks occur only locally, it is difficult to known how moisture variation could have biased the local ichnological record.

According to Mancuso et al. (2016), tracks produced in dry sand surficial layers with moist subsurfaces often preserve more detailed anatomical information. As mentioned above, the facies association of the “Pirambóia Formation” indicates a humid eolian system with the influence of ephemeral braided fluvial channels (Dias & Scherer, 2008; Rodrigues, 2014). Adhesion structures on the interdune and sand sheet deposits also indicate some influence of the high phreatic level on the deposition of the eolian strata (Rodrigues, 2014). These sedimentary structures indicate that the phreatic level was high enough to provide some quantity of moisture to the dunes, influencing its consistency. Moreover, we found raindrop marks on the sandstones from the Ibicuí d’Armada area (Fig. S3 in the Supplemental Information File), which indicates that meteoric water also contributed as a moisture source. Consequently, the Ibicuí d’Armada trackways probably were produced in dunes with definite moisture content, preserving some morphological details of the trackmakers, as can be observed on the SLIA-1 trackway.

Additionally, the trackmakers’ differences in size, body mass, and speed could have been the source of variation between the preserved tracks. There is a wide range of size among the Ibicuí d’Armada trackmakers, indicated by their inferred gleno-acetabular distances. The producer of SLIA-2 tracks was from 78.9 to 121.4 mm in length, while the SLIA-1 trackmaker could have been 408.8 mm long. Although we could not infer the gleno-acetabular length of the SLIA-3 trackmaker, its tracks indicate an even larger animal (Tables S1–S6 in the Supplemental Information File). Accordingly, besides the substrate consistency, these biological variations were the main cause of the taphonomic variability among the Ibicuí d’Armada trackways.

## Conclusion

Here, we describe from the first time five tetrapod trackways (SLIA-1 to SLIA-5) from the Lopingian–Induan “Pirambóia Formation.” The stratigraphy of this unit is unresolved for deposits that occur along the northeastern border of the Paraná Basin (in the São Paulo State), but the age of the deposits from the southwestern region of Rio Grande do Sul can be determined by their lower and upper contacts with the Rio do Rasto Formation (Guadalupian–Lopingian) and the Sanga do Cabral Formation (Induan–Olenekian), respectively.

The “Pirambóia” tracks occur in two strata (20° and 32° dip) that were part of the same dune. The lack of variation in substrate texture, mineralogical composition and bedding dip among the track-bearing strata indicates that the preservational variation among the trackways should be related to biological (e.g., size and weight of the trackmakers) or behavioral (e.g., speed) traits.

Among the described trackways, three of them are assigned to the ichnogenera *Dicynodontipus* and *Chelichnus.* While this latter ichnogenus is widespread among dune deposits of the Permian, the presence of *Dicynodontipus* in the *Chelichnus* Ichnofacies of the “Pirambóia Formation” is remarkable, because it is not often found in desert deposits. The trackway SLIA-1 (*Dicynodontipus* isp.) preserves some features that could be related to its trackmaker (e.g., size and proportion of the tracks, triangular-shaped, forward oriented *pedes* with sole pads and blunt, short digits). Comparisons with other Permo–Triassic ichnogenera and the anatomy of the autopodia of tetrapods of this age allow us to attribute SLIA-1 to dicynodonts. SLIA-2 and SLIA-5 (*C. bucklandi*) are composed of oval-shaped tracks that are here interpreted as therapsid–related. Regarding SLIA-3 and SLIA-4, we have hypothesized that they were produced by therapsids (based on their homopody and symmetry, the co-occurrence with SLIA-1 and SLIA-2, and in the age of the “Pirambóia” deposits) but no morphological details were preserved, making it difficult to make a more assertive attribution. The presence of therapsid tracks in the Lopingian–Induan of Brazil is noteworthy and fundamental to the understanding of the occupation of desert environments by tetrapods during such a crucial interval in Earth history.

## Supplemental Information

10.7717/peerj.4764/supp-1 Supplemental Information 1 Supplemental tables and figures.

## Institutional abbreviations

MLP: Museo de La Plata, La Plata, Buenos Aires Province, Argentina
MN: Museu Nacional, Rio de Janeiro, Rio de Janeiro State, Brazil
MNA: Museum of Northern Arizona, Flagstaff, Arizona, United States of America
RAM: “Raymond M. Alf” Museum of Paleontology, Claremont, California, United States of America
SLIA: non-collected track, Ibicuí d’Armada locality, Santana do Livramento, Rio Grande do Sul, Brazil
UFRGS: Universidade Federal do Rio Grande do Sul, Porto Alegre, Rio Grande do Sul, Brazil
UFRJ: Universidade Federal do Rio de Janeiro, Rio de Janeiro, Rio de Janeiro State, Brazil
USNM: United States National Museum of Natural History, Smithsonian Institution, Washington, District of Columbia, United States of America.

## Additional Information and Declarations

### Field Study Permissions

The following information was supplied relating to field study approvals (i.e., approving body and any reference numbers): The permit for field work was provided by the Departamento Nacional de Produção Mineral (DNPM, Process Number: 000.820/2015).

### Data Availability

The following information was supplied regarding data availability: The raw data are provided in a Supplemental File.
