# Supplementary material for: Tetrapod tracks in Permo–Triassic eolian beds of southern Brazil (Paraná Basin)
Source: PeerJ. 2018 May 18;6:e4764. doi: 10.7717/peerj.4764 (PMC5961629; doi:10.7717/peerj.4764)
Supplement: Supplemental Information 1 [file peerj-06-4764-s001.docx]

Supplemental information for

**Tetrapod tracks on Permo–Triassic eolian beds of southern Brazil (Paraná Basin)**

Heitor Francischini, Paula Dentzien-Dias, Spencer G. Lucas, Cesar L. Schultz

**Supplementary Tables**

**Table S1.** **Measurements of the footprints of the SLIA-1 trackway (*Dicynodontipus* isp.).**

| Footprint number | Autopodium | Width (mm) | Length (mm) | Width/Length ratio | Maximum depth (mm) | Distance from midline (mm) | Divarication from midline |
| --- | --- | --- | --- | --- | --- | --- | --- |
| I | Left *pes* | 91 | 105 | 0.86 | 36 | 70.4 |  |
| II | Right *pes* | 92 | 81 | 1.13 | 33 | 73.3 |  |
| III | Left *manus* | 81 | 49 | 1.65 | 43 | 129.6 | 24° |
| IV | Right *manus* | 60 | 42 | 1.42 | 50 | 62.3 | 37° |
| V | Left *pes* | 90 | 92 | 0.97 | 51 | 93.8 |  |
| VI | Right *pes* |  |  |  | 31 | 6.14 |  |
| VII | Left *manus* | 78 | 45 | 1.73 | 39 | 102.2 | 30° |
| VIII | Right *manus* | 74 | 53 | 1.39 | 52 | 70.0 | 35° |
| IX | Left *pes* | 74 | 95 | 0.77 | 26 | 47.7 |  |
| X | Right *pes* |  |  |  | 32 | 66.5 |  |
| XI | Left *manus* | 60 | 51 | 1.17 | 48 | 122.7 | 18° |
| XII | Right *manus* | 81 | 45 | 1.8 | 44 | 36.7 | 32° |
| XIII | Left *pes* | 85 | 59 | 1.44 | 43 | 56.2 |  |
| XIV | Right pes | 84 | 35 | 2.4 | 59 | 51.9 |  |
| Mean | *Manus* | 72.33 | 47.5 | 1.52 | 46 | 81.13 | 29.33° |
| Mean | *Pes* | 86 | 77.83 | 1.10 | 38.87 | 58.24 |  |

**Table S2. Mean values of the oblique pace length, the pace angulation, the stride length and the *manus*-*pes* distance of the SLIA-1 trackway (*Dicynodontipus* isp.).**

| Autopodium | Oblique pace length (mm) | Pace angulation | Stride length (mm) | *Manus*-*pes* distance (mm) |
| --- | --- | --- | --- | --- |
| *Manus* | 274.2 | 57.5° | 280.8 | 165 |
| *Pes* | 262.8 | 64.6° | 261.2 |  |

**Table S3.** **Mean values of the oblique pace length, the pace angulation and the stride length of the SLIA-2 trackway (*Chelichnus bucklandi*).**

| Oblique pace length (mm) | Pace angulation | Stride length (mm) |
| --- | --- | --- |
| 66.3 | 83.5° | 84.5 |

**Table S4. Measurements of the footprints of the SLIA-2 trackway (*Chelichnus bucklandi*).**

| Footprint number | Autopodium | Width (mm) | Length (mm) | Width/Length ratio | Distance from midline (mm) | Divarication from midline |
| --- | --- | --- | --- | --- | --- | --- |
| I | Left | 21 | 12 | 1.75 | 5.9 | 23° |
| II | Right | 15 | 09 | 1.66 | 15.8 | 24° |
| III | Left | 23 | 18 | 1.27 | 14.5 | 18° |
| IV | Right | 14 | 08 | 1.75 | 8.5 | 40° |
| V | Left | 16 | 10 | 1.60 | 19.3 | 18° |
| VI | Right | 22 | 16 | 1.37 | 14.0 | 18° |
| VII | Left | 26 | 15 | 1.73 | 18.1 | 20° |
| VIII | Right | 17 | 12 | 1.41 | 16.7 | 11° |
| IX | Left | 19 | 11 | 1.72 | 13.7 | 10° |
| X | Right | 19 | 11 | 1.72 | 9.4 | 23° |
| XI | Left | 15 | 08 | 1.87 | 7.6 | 16° |
| XII | Right | 15 | 07 | 2.14 | 11.5 | 31° |
| XIII | Left | 19 | 10 | 1.90 |  |  |
| XIV | Right | 17 | 06 | 2.83 |  |  |
| XV | Left | 21 | 17 | 1.23 |  |  |
| Mean | - | 18.6 | 11.3 | 1.64 | 12.9 | 21° |

**Table S5.** **Measurements of the footprints of the SLIA-3 trackway (Indeterminate).**

| Footprint number | Autopodium | Width (mm) | Length (mm) | Width/Length ratio | Distance from midline (mm) |
| --- | --- | --- | --- | --- | --- |
| I | Left | 185 | 140 | 1.32 | 123.3 |
| II | Right | 180 | 220 | 0.81 | 112.2 |
| III | Left | 110 | 90 | 1.22 | 116.8 |
| IV | Right | 196 | 215 | 0.91 | 84.4 |
| V | Left | 150 | 155 | 0.96 | 97.2 |
| VI | Right | 124 | 170 | 0.72 | 69.7 |
| Mean | - | 157.5 | 165 | 0.95 | 100.6 |

**Table S6.** **Mean values of the oblique pace length and the stride length of the SLIA-3 trackway (Indeterminate).**

| Oblique pace length (mm) | Stride length (mm) |
| --- | --- |
| 416 | 315 |

**Supplementary figures**

**
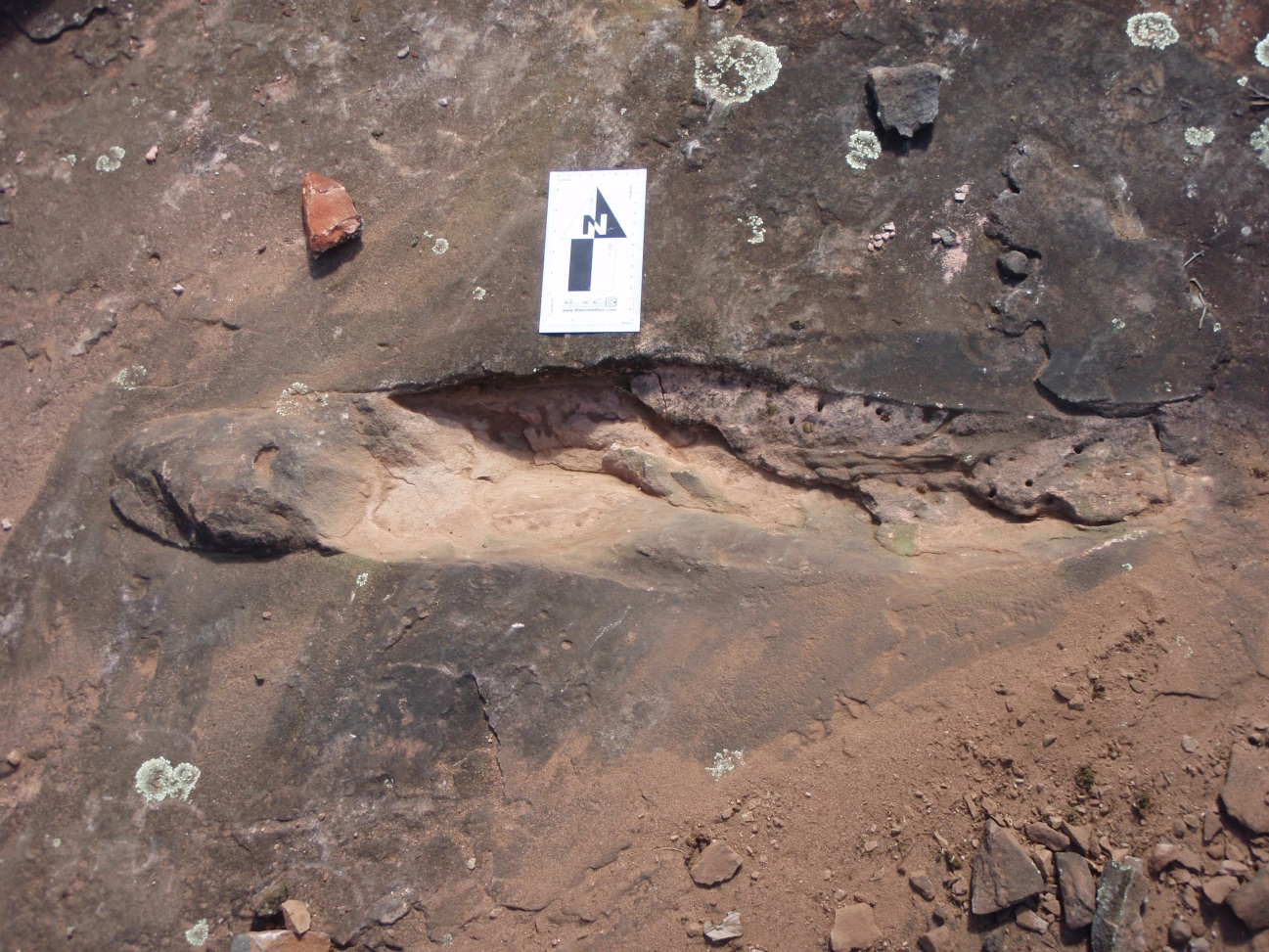
**

**Figure S1.** **Tetrapod burrow (crotovine) preserved in the same strata than SLIA-1 and SLIA-4 trackways.** Scale in centimeters. Image credit: the authors.

**
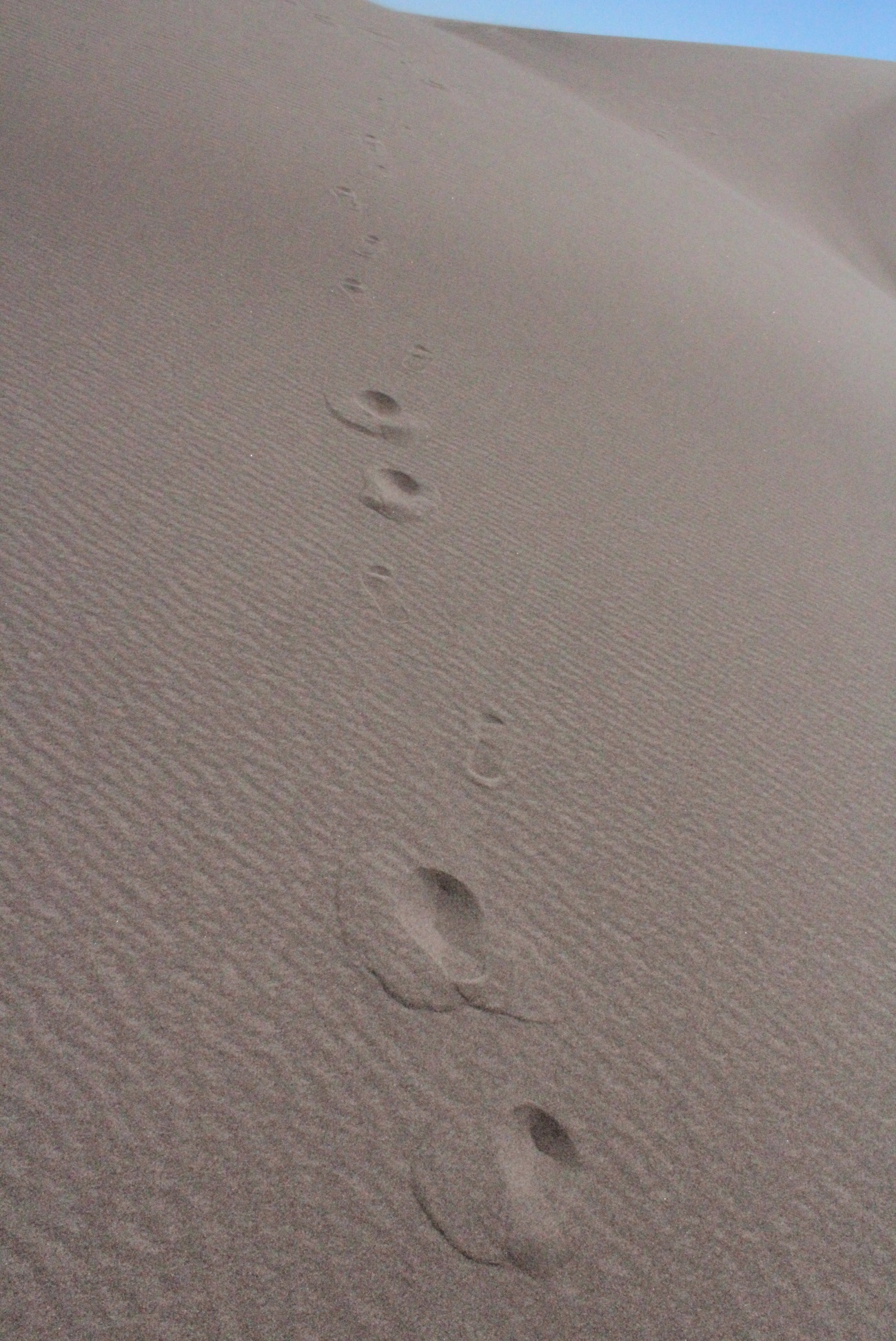
**

**Figure S2. Example of intratrackway morphological variability in eolian sand dunes.** Set of footprints produced by a human walking upslope in an eolian dune of the Great Sand Dunes National Park, Colorado, United States of America. Image credit: the authors.


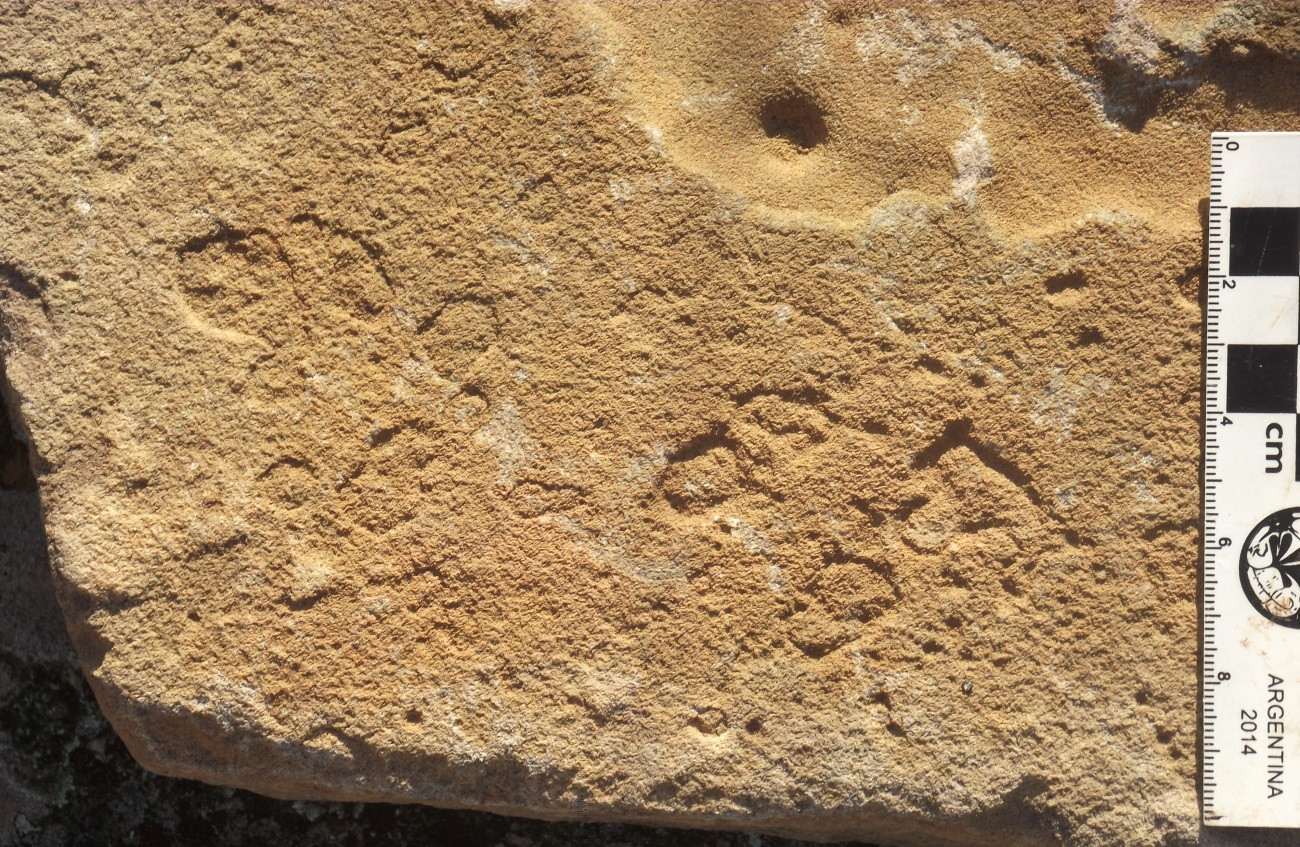


**Figure S3. Raindrop marks in an eolian bed of the “Pirambóia Formation”, Brazil.** Scale in centimeters. Image credit: the authors.
